# Probing actin‐activated ATP turnover kinetics of human cardiac myosin II by single molecule fluorescence

**DOI:** 10.1002/cm.21858

**Published:** 2024-04-16

**Authors:** Albin Berg, Lok Priya Velayuthan, Sven Tågerud, Marko Ušaj, Alf Månsson

**Affiliations:** ^1^ Department of Chemistry and Biomedical Sciences, Faculty of Health and Life Science Linnaeus University Kalmar Sweden

**Keywords:** actin, actin‐activated ATPase, Alexa 647 ATP, C2C12 cells, 1‐ethyl‐3‐(3‐dimethylaminopropyl)carbodiimide, single molecule fluorescence, β‐Cardiac myosin II

## Abstract

Mechanistic insights into myosin II energy transduction in striated muscle in health and disease would benefit from functional studies of a wide range of point‐mutants. This approach is, however, hampered by the slow turnaround of myosin II expression that usually relies on adenoviruses for gene transfer. A recently developed virus‐free method is more time effective but would yield too small amounts of myosin for standard biochemical analyses. However, if the fluorescent adenosine triphosphate (ATP) and single molecule (sm) total internal reflection fluorescence microscopy previously used to analyze basal ATP turnover by myosin alone, can be expanded to actin‐activated ATP turnover, it would appreciably reduce the required amount of myosin. To that end, we here describe zero‐length cross‐linking of human cardiac myosin II motor fragments (sub‐fragment 1 long [S1L]) to surface‐immobilized actin filaments in a configuration with maintained actin‐activated ATP turnover. After optimizing the analysis of sm fluorescence events, we show that the amount of myosin produced from C2C12 cells in one 60 mm cell culture plate is sufficient to obtain both the basal myosin ATP turnover rate and the maximum actin‐activated rate constant (*k*_cat_). Our analysis of many single binding events of fluorescent ATP to many S1L motor fragments revealed processes reflecting basal and actin‐activated ATPase, but also a third exponential process consistent with non‐specific ATP‐binding outside the active site.

## 1 INTRODUCTION

Myosins are molecular motors that produce force and motion by cyclically interacting with actin filaments under the turnover of ATP (Heissler & Sellers, 2016; Robert‐Paganin et al., 2020). The myosin II class, that underlies contraction of heart and skeletal muscle as well as non‐muscle cell motility and force development, has been most intensely studied in the myosin superfamily of motors (Heissler & Manstein, 2013). Yet, the most central aspects of its energy transduction mechanism are poorly understood, from actin‐attachment of the myosin motor domain to completion of the force‐ and motion‐generating power‐stroke (Robert‐Paganin et al., 2020), where the latter is believed to encompass a swing up to 10 nm of an integral lever arm in the motor domain structure. Unknown elements include the sequence of allosteric events that occur within the motor domain (Robert‐Paganin et al., 2020) as well as the relative timing of the power‐stroke and the release of inorganic phosphate (Pi) from the active site (Månsson et al., 2023).

To overcome the remaining knowledge gaps, new experimental systems are of interest, for example, to allow high‐throughput studies of myosin II with a range of different appropriately chosen mutations. Such developments are also increasingly important with the emergence of striated muscle myosin II as a drug target in diseases skeletal muscle (Gyimesi et al., 2020) and the heart (Cremer et al., 2022; Day et al., 2022; Spertus et al., 2021; Trivedi et al., 2020) (including the U.S. Food and Drug Administration approved drug^1^ mavacamten). Also, the related non‐muscular myosin II is of potential interest as a drug target in diseases such as cancer (Picariello et al., 2019; Trivedi et al., 2020). One of the assays that is often used in early drug screening efforts, as well as in basic studies of energy transduction mechanisms, is actin‐activated ATPase assays. In these assays, myosin motor domains or two‐headed myosin motor fragments are mixed with actin filaments and ATP in a cuvette or on a multiwell plate, where the formation of ATP breakdown products is measured. The experiments require at least a microgram of proteins (De La Cruz & Ostap, 2009), whereas transient kinetics stopped‐flow experiments require even more, in the microgram to milligram range (Walklate et al., 2019). Moreover, precious compounds to be tested, for example, for drug screening purposes, are also used in substantial quantities.

In studies of myosin function as well as in drug screening efforts, it is of interest to use myosin expressed and purified from cell systems to be able to introduce different mutations. However, striated muscle myosin II can only be expressed in active form in cultivated mouse myotubes that also express the chaperones required for proper myosin folding (Chow et al., 2002; Resnicow et al., 2010; Srikakulam & Winkelmann, 1999). The use of this expression system usually relies on the delivery of the striated muscle myosin II DNA by infecting the myotubes with transformed adenoviruses (Morck et al., 2022; Resnicow et al., 2010; Snoberger et al., 2021; Sommese et al., 2013). The use of viruses for gene delivery generates amounts of myosin that are sufficient for actin‐activated steady‐state ATPase and transient kinetics studies as well as for Cryo‐electron microscopy (Cryo‐EM). However, the procedure is costly, time consuming, and requires access to safety labs for virus handling. The method is particularly challenging to use if the study requires the generation of many different mutations because each mutation requires the generation of a new transformed virus that contains the appropriately mutated DNA. Such a procedure is expected to take around a month for each new mutation.

Recently (Velayuthan et al., 2023), we developed a method to express the cardiac myosin II motor domain that does not require viruses for gene delivery but instead relies on a polymer compound mixed with the DNA of interest. This procedure circumvents the virus generation step, thereby reducing the time for the production of a new mutation by about a month to <1 week (following gene synthesis). The procedure also overcomes other issues with the virus‐based transfection mentioned above. However, if large amounts of myosin need to be produced, for example, for stopped‐flow analyses or actin‐activated ATPase assays, key advantages of the non‐viral method are lost. This is because the production of large amounts of myosin will also require large amounts of non‐viral transfection agents with associated high costs. Therefore, in order to maximize the advantages of the non‐viral expression method (Velayuthan et al., 2023), it is important to use highly miniaturized assays that require only tiny amounts of protein. Some assays of this type already exist such as single molecule (sm) optical tweezers based mechanics (Capitanio & Pavone, 2013; Finer et al., 1994; Kaya et al., 2017; Molloy et al., 1995; Sung et al., 2017), high‐speed atomic force microscopy (hs‐AFM) structural dynamics (Ando et al., 2001; Ando et al., 2008; Moretto et al., 2022) and, finally, sm fluorescence‐based methods (Amrute‐Nayak et al., 2008; Funatsu et al., 1995; Usaj et al., 2021). The latter are most generally accessible because they can be centered around a research grade fluorescence microscope with a total internal reflection fluorescence (TIRF) accessory. The latter can be developed at appreciably less cost and efforts than hs‐AFM systems and the three‐bead optical tweezers (Finer et al., 1994) required for myosin II motors.

However, to the best of our knowledge, the only sm‐fluorescence assays that have been extensively developed so far with myosin in focus are assays for basal myosin ATP turnover using fluorescent ATP as a substrate (Amrute‐Nayak et al., 2008; Funatsu et al., 1995; Usaj et al., 2021). This should, however, be readily expanded to sm‐fluorescence resonance energy transfer (sm‐FRET) methods to detect power‐strokes suggested by similar ensemble studies in solution (Muretta et al., 2015; Shih et al., 2000; Suzuki, 2000). Recently (Usaj et al., 2021), we also studied the isometric actin‐activated ATPase of skeletal muscle heavy meromyosin (HMM) immobilized to a surface binding an actin filament at very low (ATP). This was possible due to the low ATP turnover rate under isometric conditions, 10‐ to 100‐fold lower than the actin‐activated ATPase in solution, where the myosin lever arm, in contrast to the isometric assay, is free to swing without counteracting forces. For skeletal muscle, the maximum actin‐activated activity per head (*k*_cat_) is around 50 s^−1^ at 25^o^C (Brenner & Eisenberg, 1986), corresponding to average on‐dwell‐times of the fluorescent ATP of 20 ms. Such short times are challenging to detect using standard TIRF microscopy. In contrast, *k*_cat_ of cardiac ventricular β‐myosin II is about 10 s^−1^ with a corresponding on‐dwell‐time of 100 ms (Sommese et al., 2013; Velayuthan et al., 2023). Acquisition to capture processes with such relatively slow kinetics is within the reach of the TIRF microscope. However, a problem is that the rate limiting step of the actin‐activated ATPase in solution is associated with the myosin head attachment to actin. Thus, the motor domain essentially spends the waiting time detached from actin when it diffuses around in solution. This makes it impossible to detect the bound fluorescent ATP by TIRF microscopy (which only detects immobilized fluorophores within about 100 nm from the surface). A way to potentially overcome this problem would be if myosin can be cross‐linked to actin with maintained actin‐activated ATPase. Indeed, there is one such method available (Brenner & Eisenberg, 1986; Huang et al., 1990; Mornet et al., 1981; Stein et al., 1985) that has been used for previous ensemble studies with myosin motor domains and actin in solution, for example, to measure *k*_cat_ at high ionic strength when the actin affinity is otherwise low. However, this method has not previously been applied to sm studies.

Here, we report utilization of the abovementioned cross‐linking approach (Brenner & Eisenberg, 1986; Huang et al., 1990; Mornet et al., 1981; Stein et al., 1985) for measuring *k*_cat_ of actin‐activated ATPase (solution type, with the lever arm free to swing; Craig et al., 1985) of cardiac ventricular myosin motor domains expressed in, and purified from, C2C12 cells. We show that enough cardiac myosin S1 for our analyses is obtained from one 6 cm cell cultivation plate following virus‐free transfection. The results show that the cross‐linking approach can be used in a sm setting and only requires nanogram quantities of myosin, that is, almost 1000‐fold less than solution‐based assays and with the possibility to reduce the sample volumes to a few microliters. This will allow fast and cheap drug screening and studies where energy transduction mechanisms can be systematically and comprehensively interrogated by subjecting myosin to many different mutations, challenging to accomplish using standard large‐scale virus‐based transfection systems.

## 2 MATERIALS AND METHODS

### 2.1 Materials and chemicals

2‐(N‐Morpholino)ethanesulfonic acid hydrate (4‐morpholineethanesulfonic acid, MES Hydrate) was from Sigma‐Aldrich, (cat no. M8250) and 1‐ethyl‐3‐(3‐dimethylaminopropyl)carbodiimide hydrochloride (EDC) from Thermo Scientific (cat. no. 22980). Specialized assay solution components include pyranose oxidase (POX) from Sigma‐Aldrich (cat. no. P4234), cyclooctatetraene (COT) from Sigma‐Aldrich (cat. no. 138924), 4‐nitrobenzyl alcohol (NBA) from Sigma‐Aldrich, (cat. no. N12821), Trolox from Sigma‐Aldrich (cat. no. 238813), creatine phosphate (CP) from Sigma‐Aldrich (cat. no. P7936) and Creatine phosphokinase (CPK) from Sigma‐Aldrich (cat.no. C3755). Bovine serum albumin (BSA) of high purity was obtained from Sigma‐Aldrich (cat. no. A0281) whereas BSA of standard purity was from Sigma‐Aldrich (cat. no. A2153). Streptavidin was from Sigma‐Aldrich (cat. no. S4762). Substances for labeling actin filaments were from Invitrogen, including biotin phalloidin: (cat. No. B7474), rhodamine‐phalloidin: (cat. No. R415) and Alexa 647‐phalloidin: (cat. no. A22287). All other chemicals used were of analytic grade and obtained from Sigma‐Aldrich or Thermo Fisher.

### 2.2 Proteins

Actin was purified from rabbit back skeletal muscle following the protocol of Pardee and Spudich (1982). Myosin II was purified from hind leg white skeletal muscle (also from the rabbit) for further preparation of the motor fragment, HMM. Myosin purification and HMM preparation were performed based on procedure originally described by Margossian and Lowey (1982) and modified as in Sata et al. (1993) and Kron et al. (1991). N‐ethyl maleimide treatment of HMM (NEM‐HMM) makes HMM‐binding to actin filaments insensitive to ATP. The NEM‐HMM was produced by treatment of HMM with NEM as described previously (Bengtsson et al., 2016) (see also references therein).

The rabbits for preparation of skeletal muscle actin and myosin were euthanized in accordance with appropriate legislation as approved by the Regional Ethical Committee for Animal Experiments in Linköping Sweden (ref. 17088‐2020). The rabbit was anesthetized by an intramuscular injection of zolazepam 6 mg/kg, tiletamin 6 mg/kg, and medetomidine .6 mg/kg immediately before euthanization by injection of 2 mL of penthobarbital (100 mg/mL) in an ear vein. All procedures were performed by the Linnaeus University veterinarian.

The cardiac β‐myosin II motor domain (myosin heavy chain; MYH7 gene) fused to enhanced green fluorescent protein (eGFP), with a FLAG tag for purification, was expressed in C2C12 mouse myotubes. The plasmid construct, virus‐free transfection, expression and purification of human β‐cardiac myosin (sub fragment 1‐long) are described in detail previously (Velayuthan et al., 2023). In brief, a truncated MYH7 gene consisting of 1–848 amino acid (“sub‐fragment 1 long”; S1L) was fused to a Tobacco etch virus protease site in sequence with eGFP and a FLAG tag at the C‐terminal. It was then cloned into pcDNA‐3.1 plasmid under the cytomegalovirus (CMV) promoter (GenScript Biotech Corporation). The plasmid was transiently transfected (non‐virally) into C2C12 myoblast cells using the JetPrime® DNA transfection reagent kit and allowed to differentiate into myotubes for 7 days. On Day 7, the cells were imaged to visualize protein expression via the eGFP tag, using an inverted fluorescence microscope (Axio Observer D1, Zeiss, Germany). Later, the cells were harvested to purify expressed proteins from a single 6 cm cell cultivation plate by miniaturizing our protein purification protocol. The expressed human β‐cardiac myosin was co‐purified with mouse regulatory and essential light chains using Anti‐FLAG resin® M2 affinity gel (Sigma‐Aldrich). In many basic studies of motor functions, it is not necessary to exchange the light chains for cardiac isoforms whereas this may be important in studies of cardiac diseases and drugs.

The final eluate was filtered to remove residual resin using Pierce micro‐spin columns (Thermo Scientific). The remaining ATP from the protein purification was eliminated by dialysis thrice against a volume of 125 mL X‐linking wash buffer (10 mM imidazole, 5 mM KCl, 3 mM MgCl_2_, 1 mM dithiothreitol (DTT), pH 7.5) a buffer that was also used in connection with the EDC cross‐linking (“X‐linking”) of S1L to actin. BSA, 1 mg/mL (final concentration) was also added to the protein sample upon dialysis. Dialysis was performed in a Harvard apparatus Fast Spin Dialyzer (chamber volume 200 μL) with regenerated cellulose membrane having a molecular weight cut‐off of 10 kDa.

Cross‐linking and ATPase assay experiments were performed within 24 h of protein purification. During that time, the protein sample was dialyzed into the X‐linking wash buffer and then kept on ice in a cold room. Expressed β‐cardiac myosin S1L, including the eGFP‐Flag C‐terminal extension will be denoted as “S1L” below.

### 2.3 Labeling of F‐actin with phalloidin and phalloidin conjugates

A tube of F‐actin, stored at −80 °C, was thawed 1 h prior to the labeling. For double labeling of actin filaments with biotin phalloidin and rhodamine‐phalloidin or Alexa 647‐phalloidin, the final concentration of labeled phalloidins was 3 μM each. For single dye labeling (or just plain phalloidin labeling), the final concentration of labeled phalloidin (or plain phalloidin) was 6 μM. The final concentration of actin filaments (on a monomer basis) was 4 μM. A labeled actin filament stock solution was prepared at a final volume of 200 μL in actin filament labeling buffer (60 mM KCl, 1 mM MgCl_2_ in low ionic strength solution [LISS; see below] buffer pH 7.4). First, the volume of methanol into which the phalloidin stock conjugates were dissolved was removed by evaporation using gentle N_2_ gas flow before diluting in actin filament labeling buffer mixed with a thawed stock vial of actin filaments (50–100 μM on monomer basis). The prepared labeled actin filaments were stored on ice in the dark. The LISS mentioned above contained 1 mM MgCl_2_, 10 mM MOPS (3‐(N‐morpholino)propanesulfonic acid, 4‐morpholinepropanesulfonic acid), and .1 mM K_2_ ethylene glycol‐bis(β‐aminoethyl ether)‐n,n,n′,n′‐tetraacetic acid.

### 2.4 Expression analysis and protein characterization

Western blotting was used to detect purified S1L given that the concentration is below the detection threshold of sodium dodecyl sulfate polyacrylamide gel electrophoresis. Proteins were combined with Pierce™ LDS non‐reducing sample buffer (Thermo Fisher) and 50 mM DTT followed by heating the mixture at 95 °C for 5 min. The samples were loaded on to a pre‐casted NuPAGE™ 4–12% Bis‐Tris gel (Thermo Fisher) and electrophoresis was performed in a cold room (4 °C) at 90 V for 45 min with a subsequent increase to 140 V for an hour in NuPAGE™ MES SDS running buffer (Thermo Fisher). Using Trans‐Blot transfer system (BioRad), the proteins were transferred to .2 μm polyvinylidene fluoride membrane at 1A and 25 V for 30 min. The membrane was incubated in .2% blocking buffer (EZ block, Biological Industries) for an hour and washed with Tris‐buffered‐saline with Tween (TBS‐T buffer; 20 mM Tris, 150 mM NaCl, .1% Tween 20, pH 7.4–7.6). Then, it was incubated with anti‐FLAG antibodies (ab1257 Abcam; 1:20,000 dilution in blocking buffer) overnight at 4 °C. The following day, the membrane was washed with TBS‐T, incubated with donkey anti‐goat antibodies (ab6885 Abcam; 1:20,000 dilution in TBS‐T) for an hour at room temperature (RT) and subjected to a final wash with TBS‐T before developing the membrane using Novex™ ECL Chemiluminescent substrate kit (Thermo Fisher). Bands were visualized in the ChemiDoc XRS gel imaging system.

### 2.5 Microscopy

#### 2.5.1 Epifluorescence microscopy

For initial studies of F‐actin (Rhodamine‐ and Alexa 647 phalloidin labeled) immobilized on nitrocellulose surfaces, images were acquired using a Hamamatsu Electronmultiplication charged coupled device (EM‐CCD) Digital Camera C9100 controlled by the HCImage software at gain 200 and 200 ms exposure time. The camera was attached to a microscope (Zeiss observer D1) equipped with a 63× magnification objective (NA 1.4) and Cy3 filter cube (Ex 532.5–557.5, EM 570‐640, DM 565). For capturing images of transfected and differentiated C2C12 cells, gain 100 and 180 ms exposure times were used instead, together with a 10× magnification objective and fluorescein isothiocyanate (FITC) filter cube (Ex 465–495 Em 515–555, DM 505).

#### 2.5.2 TIRF‐microscopy

**FIGURE 1:**
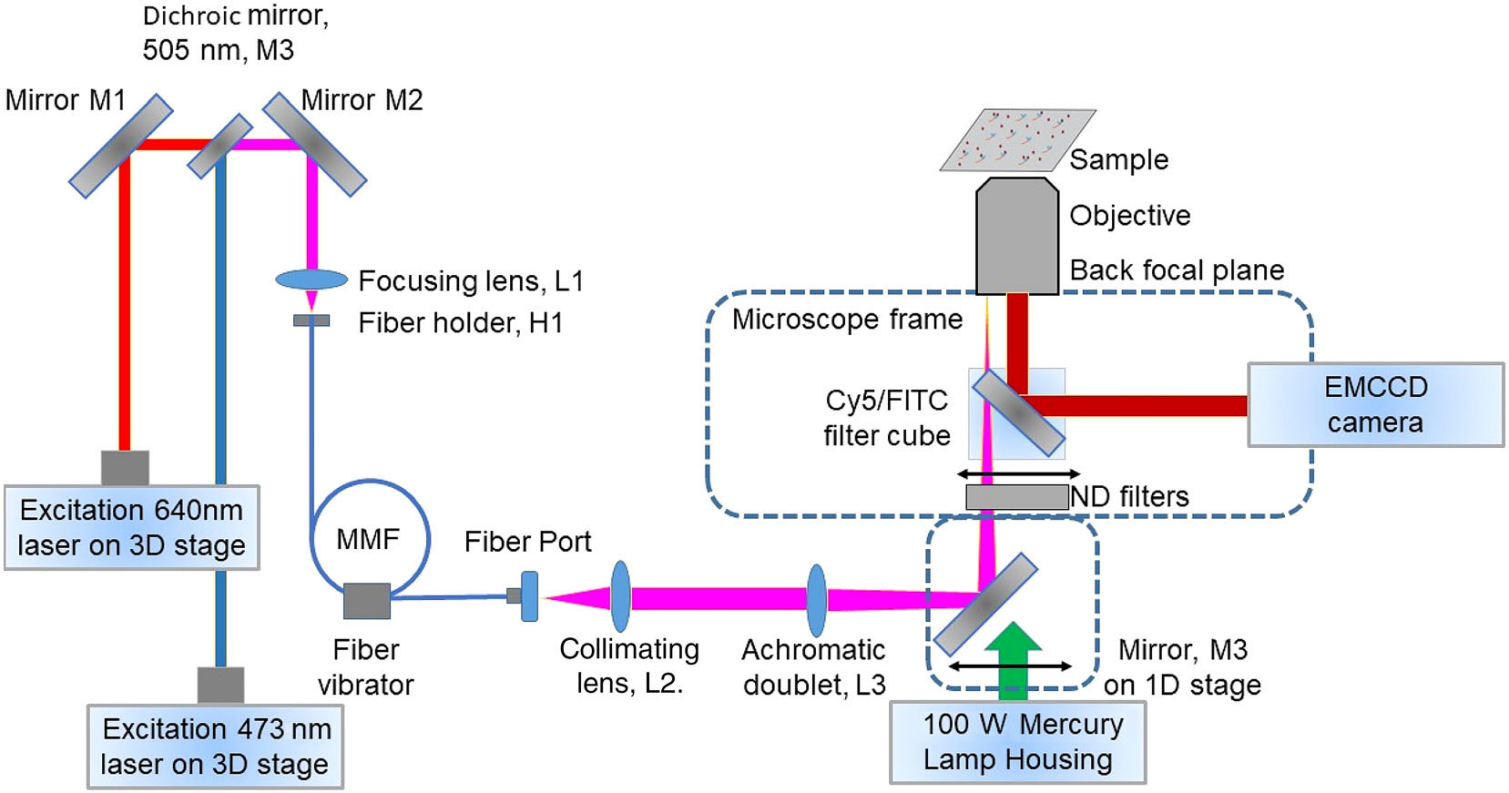
Schematic illustration of set‐up for dual laser total internal reflection fluorescence. FITC, fluorescein isothiocyanate, MMF, multimode fiber.

The Alexa 647‐ATP binding was observed using our in house built TIRF microscope (Figure 1) equipped with a 60× oil immersion objective (NA 1.49), Cy5 filter cube (Ex 590–650 Em 662.5–737.5, DM 660) and a red laser, (Melles Griot 05‐LHP‐925 30 mW, 632.8 nm HeNe laser). For observation of eGFP, we instead used a blue laser (Changchun New Industries Optoelectronics Tech. Co, Ltd, Blue Solid‐State Laser, MLL473, 473 nm, 50 mW) and a FITC filter cube. The videos were recorded using an electronmultiplication charged coupled device camera Andor iXon Ultra 897 controlled by NIS Elements software (Nikon, ver. 4.51) with gain set at 100 and exposure time at 50 ms). The setup is described in depth in Usaj et al. (2021).

### 2.6 Flow cells and glass surface treatment

A high precision cover glass (Nordic biolabs, 24 × 50 mm, 170 ± 5 μm No. 1.5 H) was used for the TIRF microscopy assays. For studies using epifluorescence microscopy, 24 × 60 mm #0 Menzel‐Gläser cover glasses were used instead. The cover glasses were cleaned by air plasma ashing (plasma cleaner Femto Standard Diener electronic GmbH, Germany) at 100 W (40 kHz), 0‐6‐.8 mbar pressure for 3 min before use. This specific method of glass slide cleaning has previously been shown to effectively remove any unidentified fluorescent objects on the cover glass, which could interfere with the sm assay (Usaj et al., 2021).

For the deposition of nitrocellulose on the cover glass, an equal amount of 2% parlodion in amyl acetate (cat. 12620‐50 Electron Microscopy Sciences) and amyl acetate (cat. 10815 Electron Microscopy Sciences) was mixed followed by 30 min incubation at RT (20–23 °C). Only a volume of 5 μL of the mixture is necessary to coat one cover glass. The mixture is added to the cover glass surface and smeared out by dragging another cover glass held to the deposited droplet at 45° angle.

The flow cell was created by cutting double sided tape (3M Scotch) into halves that were then stuck to the nitrocellulose treated cover glass delimiting an area of 2 mm in width and 2 cm in length. A square cover glass (18 × 18 mm) was then placed on top of the tape to create a chamber where 10 μL of liquid can be held. Ethanol‐cleaned scissors and tweezers were used to minimize the introduction of unidentified particles while creating the chamber.

### 2.7 Actin filament immobilization on nitrocellulose‐coated glass surfaces

Actin filaments were immobilized to nitrocellulose‐coated glass surfaces by different approaches, as described below. For each specific method, a final concentration of .1–1 mg/mL standard purity BSA (high purity used for TIRF experiments) was added after immobilization of F‐actin on the surface but before infusion of S1L.

#### 2.7.1 Via NEM‐HMM

NEM‐HMM was diluted to 37.7 μg/mL in wash buffer (50 mM KCl, in LISS, pH 7.4). The NEM‐HMM was then added to the chamber with incubation for 5 min followed by rinsing with wash buffer. Rhodamine phalloidin labeled F‐actin (10 nM) was then added to the chamber and incubated for 2 min followed by two washes.

#### 2.7.2 Via biotin–streptavidin

Streptavidin (1 mg/mL, in wash buffer defined above) was added to the nitrocellulose chamber, followed by a brief incubation (2 min) and rinsing with wash buffer. Biotin/rhodamine‐phalloidin‐labeled F‐actin was then introduced, followed by incubation for 5 min.

#### 2.7.3 Via EDC cross‐linking

F‐actin was cross‐linked to nitrocellulose, inspired by information in https://tools.thermofisher.com/content/sfs/brochures/1602163-Crosslinking-Reagents-Handbook.pdf. The conditions for this cross‐linking were based on studies where myosin was cross‐linked to actin (Mornet et al., 1981; Stein et al., 1985), but with slight modifications. F‐actin (labeled with phalloidin) at a concentration of 20 nM in X‐linking wash buffer (10 mM imidazole, 5 mM KCl, 3 mM MgCl_2_, 1 mM DTT, solution pH adjusted to 7.5 with KOH) was added to the nitrocellulose‐coated surface in the flow cell followed by incubation for 1 min. A concentration of 15 mM EDC crosslinker in 50 mM MES pH 6.5, 1 mM DTT was then added to the chamber to activate the carboxylic groups in F‐actin leading to the attachment of F‐actin to the nitrocellulose surface. The cross‐linking reaction was carried out for 1 min and quenched by rinsing the chamber with wash buffer three times.

### 2.8 EDC cross‐linking of S1L to F‐actin

The MES and X‐linking wash buffers (see above) were employed. Both were degassed for 30 min and sterile filtered (.2 μm) before use.

To promote rigor binding of S1L to actin and thereby also reducing unspecific surface adsorption of free S1L, it was important to remove ATP from the purified S1L sample by dialysis as described above. This is also crucial for cross‐linking in the correct nucleotide‐dependent conformation (Arata, 1984).

The nitrocellulose surface with immobilized F‐actin was blocked by adding 1 mg/mL of BSA in X‐linking wash buffer. Subsequently 5 μL, out of 200 μL (total elution volume) of the purified myosin S1L from one 60 mm cell cultivation plate, also containing 1 mg/mL BSA, was added to the chamber with the excess withdrawn slowly. The attachment of S1L to F‐actin was observed using TIRF microscopy, allowed by the fused eGFP. Once attachment was visibly confirmed (<2 min), molecular cross‐linking of actomyosin was performed by introducing 15 mM EDC crosslinker (50 mM MES pH 6.5, 1 mM DTT) to the rigor actomyosin complex. The reaction was then allowed to proceed for 10 min. Quenching of the cross‐linking reaction was performed by rinsing with X‐linking wash buffer four times. Addition of 50 nM cold ATP (non‐fluorescent ATP) in X‐linking wash buffer removed un‐crosslinked S1L by dissociation from actin. Moreover, it blocked the active sites of any non‐functional S1L molecules that did not turn over ATP (cf. Amrute‐Nayak et al., 2008; Usaj et al., 2021). Blocking of the surface to prevent myosin head adsorption was achieved by including .5 mg/mL BSA (final concentration) in the X‐linking wash buffer with cold ATP. A sequence of three new washes, using X‐linking wash buffer, was then performed. Finally, a volume of two to three droplets of assay solution (see below) was added from a syringe followed by sealing of the chamber with vacuum grease.

### 2.9 Single molecule ATP turnover assay and recording of on‐dwell events

Our optimized TIRF assay solution (denoted “assay solution” in the following) is based on that in Usaj et al. (2021), as briefly outlined below.

Trolox was prepared fresh in LISS buffer (see above) at a final concentration of ~2 mM. The Trolox was first dissolved in methanol to a 100 mM solution and was thereafter further diluted in LISS buffer. The pH was adjusted to 7.4 by KOH titration. The solution was filtered (.2 μm) into a 10 cm Petri dish and subsequently exposed to UV‐light (254 nm) to form Trolox‐Quinone. The solution was exposed for 15 min (120,000 μJ/cm; Heissler & Sellers, 2016) using stratlinker1800 (Stratagene). This procedure yielded approximately 20% Trolox‐Quinone (molar ratio).

The assay solution was then prepared in Trolox/Trolox‐Quinone‐LISS containing 45 mM KCl, 10 mM DTT, 7.2 mg/mL glucose, 3 U/mL POX, .01 mg/mL catalase, 2.5 mM CP, .2 mg/mL CPK, 2 mM COT, 2 mM NBA, ~2 mM Trolox/Trolox‐Quinone, and .64% methylcellulose with Alexa 647‐ATP (final concentration 5 nM).

The components of the TIRF assay solution have been optimized (Usaj et al., 2021), to stabilize the fluorophore photophysics. Such stabilization, includes minimized photoblinking and photobleaching (Usaj et al., 2021) with the photobleaching rate constant as low as .004 s^−1^. Importantly, this low rate would largely eliminate any disturbances to our measurements because it is an order of magnitude lower than the rate constants associated with myosin Alexa nucleotide bindings observed in the present study.

The assay solution also contained 1 mg/mL high purity BSA and, in some cases 500 nM cold (non‐fluorescent) ATP. No cold ATP was used when the main aim was clear visualization of Alexa 647 ATP binding over time. It was used, however, to facilitate the detection of individual Alexa 647 ATP binding events at high S1L density. The ionic strength of the assay solution was 60 mM and the temperature during the recordings of turnover was 23 ± 1 °C (range).

Cardiac myosin binding of Alexa 647 nucleotide to S1L on surface‐immobilized F‐actin, was recorded for 5–15 min using a frame‐to‐frame interval of 52 ms and an individual exposure time of 50 ms (Usaj et al., 2021).

After recording of the video, a Z‐projection was created using Image J (Fiji) to visualize either the average pixel values or the standard deviation of the pixel values (as indicated below) for many individual frames. The method was used to highlight specific data/pixels from the stack and was accessed *using Image › Stacks › Z‐Projection*.

### 2.10 Selection of events for analysis of actin‐activated ATP turnover

Regions chosen for analysis were selected by overlapping Alexa 647 ATP Z‐projections (standard deviation projection) with corresponding myosin eGFP Z‐projections (average intensity projection) of the same region. Once the regions with overlapping Alexa 647 ATP and eGFP projections had been identified, individual regions of 3 × 3 pixels (.8 × .8 μm^2^) along the filament were studied by using the ImageJ function *Image › Stacks › Plot Z‐axis Profile*. This allows detection of the fluorescence intensity variation throughout a video sequence in a specific region of interest (ROI) containing one or very few myosin molecules with the goal of only one Alexa 647 ATP binding event at a time. The data were analyzed based on a series of criteria. We first decided to exclude recordings with high background content estimated as appearance of >10 fluorescence events per 10 min occurring in the 3 × 3 pixels^2^ ROI in the background outside filaments. The reason for such high background in some recordings is not fully understood but could be related to occasional highly contaminated glass slides.

After the above selection step where whole recordings could be excluded, the criteria for selecting individual fluorescent Alexa 647 nucleotide binding events for analysis can be summarized as follows: (i) Observed events overlapping with GFP fluorescence appearing to be on a filament (co‐localization with eGFP and filament), (ii) single‐step change in intensity at start and end of event, (iii) higher intensity of an event than a given threshold, defined as at least twice the background noise level (defined in Section 3), and (iv) more than 15 events in total at the location studied during an entire recording and events occurring throughout the recorded time.

### 2.11 Quantitative analysis of Alexa 647 ATP turnover data

The collected dwell times were plotted in the form of a cumulative distribution as previously described (Amrute‐Nayak et al., 2014; Usaj et al., 2021) that was then fitted by a double or triple‐exponential function using nonlinear regression in GraphPad Prism (v 10). The estimated rate constants and the relative amplitude of the fast process are represented below as mean ± 95 confidence interval (95% CI) obtained in the fits.

### 2.12 Analysis of the density of S1L cross‐linked to F‐actin

Because the S1L protein material, purified from one single 6 cm plate, is not enough to reliably determine the concentration with traditional methods, we estimated the myosin density of F‐actin after cross‐linking. The mean eGFP light signal (gray scale value) emitted from one single myosin molecule (isolated dot) was estimated by studying five individual S1L molecules randomly adsorbed to the surface outside the actin filament. The minimum gray scale value (background) was also subtracted. The intensity along a filament of interest was then estimated by a similar approach (average grayscale value × area − background × area). The calculated number was divided by the value estimated from one single eGFP molecule, giving the number of eGFP molecules per F‐actin. The F‐actin length (μm) was measured and multiplied by 362 (number of actin monomers/μm) (e.g., Hild et al., 2010). The fraction of actin monomers bound to myosin (detected by eGFP) was then estimated by dividing the number of eGFP molecules per F‐actin with the total number of actin monomers in the filament. This procedure was executed using the Image J analyze feature.

### 2.13 BASAL MYOSIN ATPase

**FIGURE 2:**
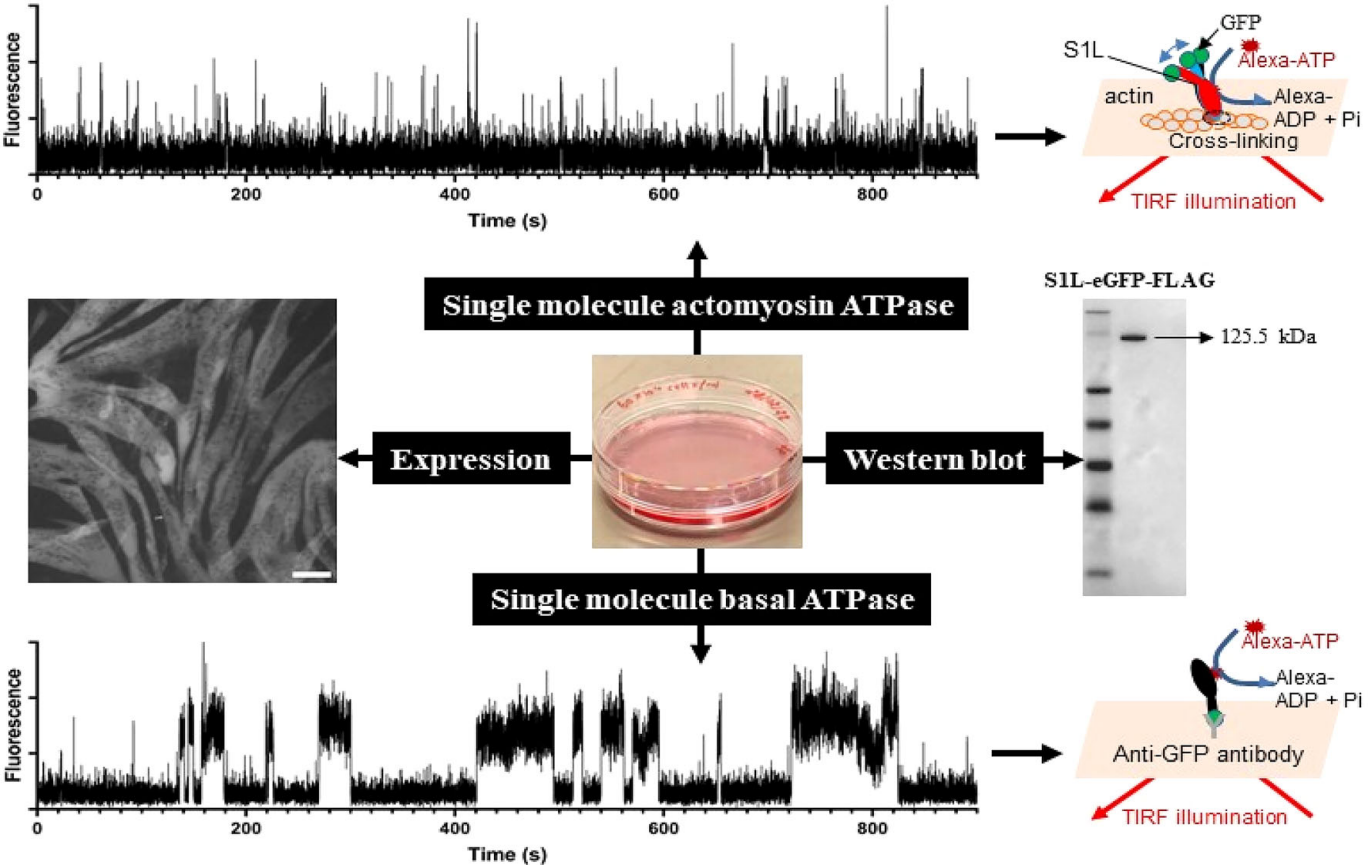
Experimental principles. eGFP, enhanced green fluorescent protein. "GFP" used instead of "eGFP" in schematic figures but with the same meaning.

The actin‐activated ATPase traces were compared to basal ATPase (method developed in Usaj et al., 2021; Velayuthan et al., 2023) performed in separate experiments. In brief, the expressed SIL was immobilized by capturing via anti‐GFP antibodies adsorbed nitrocellulose (Figure 2). After introduction of an assay solution containing 5 nM Alexa 647 labeled ATP, binding events that co‐localized with eGFP fluorescence were recorded and used to estimate basal ATPase as described previously (Usaj et al., 2021; Velayuthan et al., 2023).

### 2.14 Monte‐Carlo simulations

We simulated idealized sm recordings (without noise or other complexities) using a Monte‐Carlo simulation approach to elucidate different factors that may affect the analysis. This includes the observed percentage fractions (amplitudes) of simultaneous fast and slow events, the uncertainties introduced by recording a limited number of Alexa‐ATP turnover events and the effects on the observed rates of using different given camera frame rates and different threshold intensity values for selecting data for analysis. The simulations were performed assuming that two different processes (associated with myosin motor domains with different properties) may occur in each ROI. This is a reasonable assumption because the ROIs that we analyzed were composed of 3 × 3 pixels, corresponding to .8 × .8 μm^2^. In the Monte‐Carlo simulations we assumed, for simplicity, the presence of one myosin motor domain with fast Alexa 647 ATP binding (rate constant .005 nM^−1^ s^−1^) and fast turnover (8 s^−1^; e.g., an actin‐associated molecule) and one motor domain with slow ATP binding (rate constant .002 nM^−1^ s^−1^) and slow turnover (.05 s^−1^; e.g., a myosin motor domain not accessible for actin binding). The on‐rate constant values were chosen arbitrarily to give well separated events at time intervals like those seen in real recordings and with very rare occurrence of overlapping events. However, interestingly, the chosen values turn out (without explicit intention) to be very similar to the corresponding values (.0028 and .0048 nM^−1^ s^−1^) found recently (Usaj et al., 2021) for Alexa 647 ATP binding to fast skeletal muscle myosin and actomyosin, respectively. The simulations were implemented using the Gillespie algorithm (Gillespie, 1976; see also Mansson, 2016). Further details are given in the Supporting Information.

## 3 RESULTS

The principle of our experimental system is illustrated in Figure 2. Briefly, actin filaments are immobilized on a surface by EDC cross‐linking described above. The myosin motor fragment fused to eGFP and FLAG peptide (S1L) is expressed in C2C12 cells cultivated in a 6 cm plate and then purified from these cells 7 days after transfection, Next, S1L is cross‐linked to the actin filament using the zero‐length cross‐linker EDC. The cross‐linked S1L can be observed using sm TIRF‐based fluorescence due to its fused eGFP. Alexa 647 ATP turnover is then analyzed from sm events where the Alexa 647 ATP fluorescence co‐localizes with the eGFP fluorescence on the filament. Each component of the experimental system is considered in detail below.

### 3.1 Analysis of S1L expression

**FIGURE 3:**
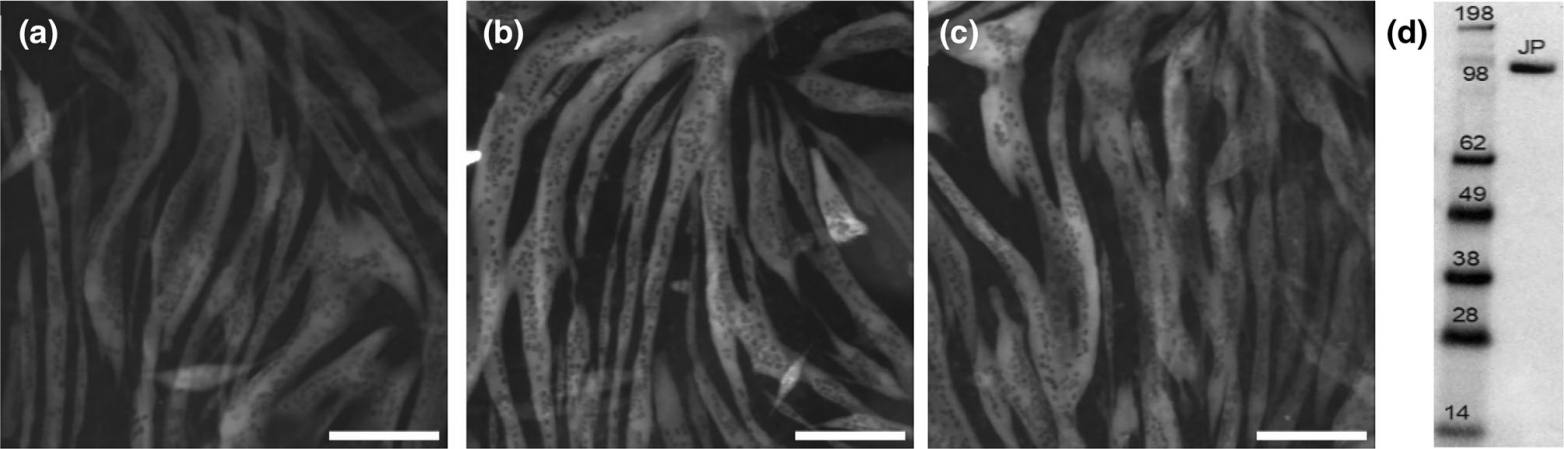
Expression of sub‐fragment 1 long (S1L) following transfection of C2C12 cells. (a–c) Three representative areas of 60 mm cell culture plates, showing transfected C2C12 cells expressing the S1L protein 7 days post‐transfection. S1L visualized by observing the fused eGFP in the epifluorescence microscope. (d) Western blot of purified protein from one single 60 mm cell culture dish using anti‐Flag antibodies for staining. Numbers presenting marker size (kDa) while “JP” refers to JetPrime S1L. Scale bar, 200 μm.

C2C12 cells in 60 mm culture plates that express S1L following the non‐viral transfection protocol (Velayuthan et al., 2023) are illustrated in Figure 3 together with a Western blot of the purified protein.

### 3.2 Immobilization of actin filaments on nitrocellulose‐coated coverslip

**FIGURE 4:**
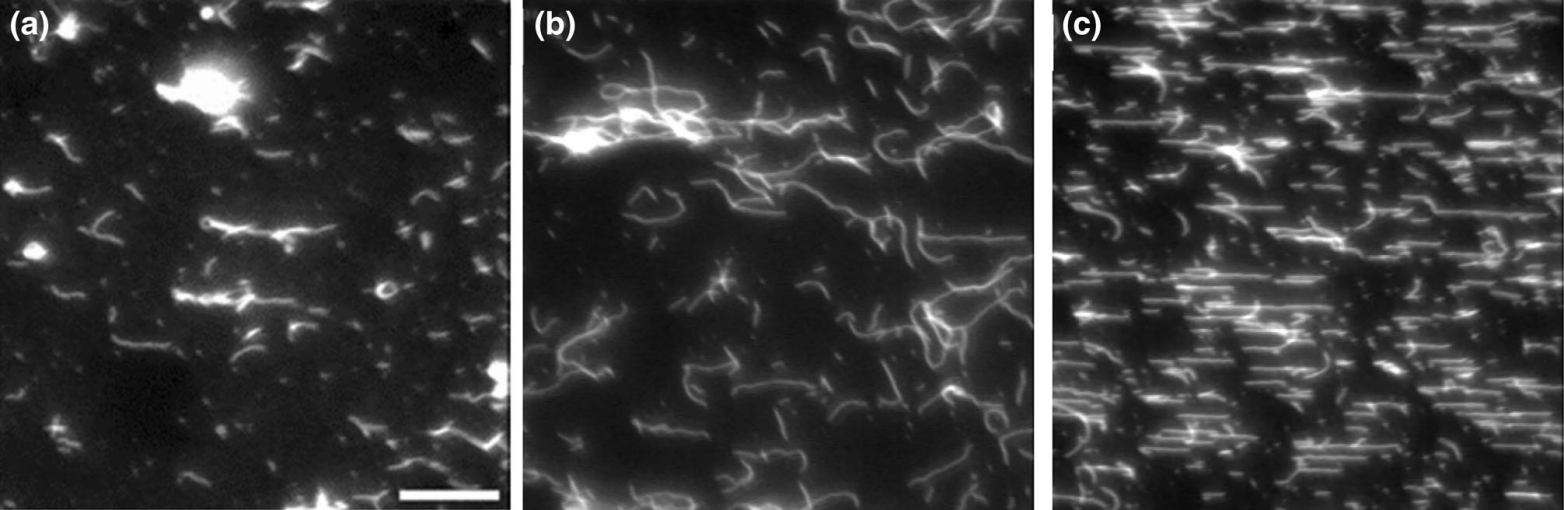
Surface immobilization of actin filaments. (a) Streptavidin biotin attachment. Biotin–streptavidin‐based attachment immobilized the rhodamine/biotin‐phalloidin labeled F‐actin on the surface (b) N‐ethyl maleimide‐heavy meromyosin attachment. (c) Cross‐linking of F‐actin to nitrocellulose using EDC cross‐linker. Picture brightness adjusted in Image J for similar intensity of individual filaments in each panel. Scale bar, 20 μm. See also Movies [Link], [Link].

Three different methods of immobilizing the actin filaments were tested (Figure 4; Movies [Link], [Link]). Repeated tests (>5 for each method) revealed that more consistent and firmer attachment of actin filaments was observed using immobilization via NEM‐HMM or by cross‐linking F‐actin directly to nitrocellulose in comparison to immobilization via streptavidin–biotin. Whereas initially firm attachment of biotinylated actin filaments was achieved with surface‐adsorbed streptavidin, subsequent addition of BSA (.1–1 mg/mL) weakened the binding and F‐actin detached from the surface over time. We attribute this to surface competition between streptavidin and BSA. The surface immobilization via NEM‐HMM was firmer and unaffected by BSA. However, despite being largely ATP insensitive NEM‐HMM heads were still binding Alexa 647 ATP (Figure S1). Because NEM‐HMM was initially adsorbed over the entire surface, such binding caused high fluorescence background noise and Alexa 647 ATP depletion. Direct cross‐linking of F‐actin to nitrocellulose using the EDC cross‐linker was not associated with the drawbacks of attachment via NEM‐HMM or biotin–streptavidin. Thus, the method resulted in consistent and stable immobilization that was not affected by BSA addition. Moreover, no extra source of background fluorescence was introduced as was seen with NEM‐HMM.

### 3.3 Cross‐linking of S1L to F‐actin

Our initial intention was to cross‐link S1L to surface‐immobilized actin filaments in a configuration where individual myosin motor fragments could be distinguished to enable studies of the actin‐activated ATP turnover from one motor fragment at a time. However, we found that cross‐linking of cardiac myosin S1L to give low density decoration as required to consistently observe individual sm was not possible under the conditions we have tested so far. When very few myosin motors were decorating the F‐actin filaments this seemed to be correlated with a high random myosin adsorption to the surface outside the filaments. This made it challenging to reliably distinguish the filamentous pattern of myosin, based on eGFP fluorescence, above the background. Observations of the events, from initial binding of S1L to actin in rigor to completed cross‐linking and rinsing, gave insight into the basis for the problems. Thus, we found that any introduction of new solution (crosslinker, MES or X‐linking wash buffer) resulted in S1L detachment particularly when S1L was used in low concentrations with the aim to achieve low S1L density along the filaments. The detached S1L molecules tended to adsorb to the surface contributing to noticeable background signals at low S1L concentrations. We further discuss the possible basis for these problems and their relevance, below.

**FIGURE 5:**
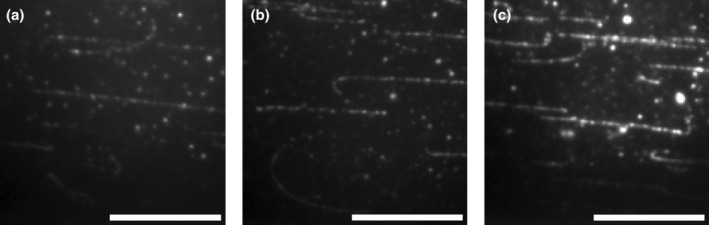
Variation in sub‐fragment 1 long (S1L) decoration and unspecific surface adsorption following cross‐linking of S1L to phalloidin labeled actin filaments. (a–c) Three representative fluorescence microscopy images showing the enhanced green fluorescent protein (eGFP) fluorescence from three separate protein purifications. Cross‐linking with 15 mM EDC for 10 min followed by addition of 50 nM non‐fluorescent ATP. Undiluted S1L directly derived from the protein preparations was used in these experiments. The background is subtracted with the rolling ball algorithm (rolling ball radius: 5 pixel) in image J. Note that the intensity of individual eGFP associated dots is on a similar level in panels (a–c). Therefore, the apparently different intensity between the panels clearly indicates the difference in the density of cross‐linked proteins between the experiments. Scale bars, 20 μm.

**FIGURE 6:**
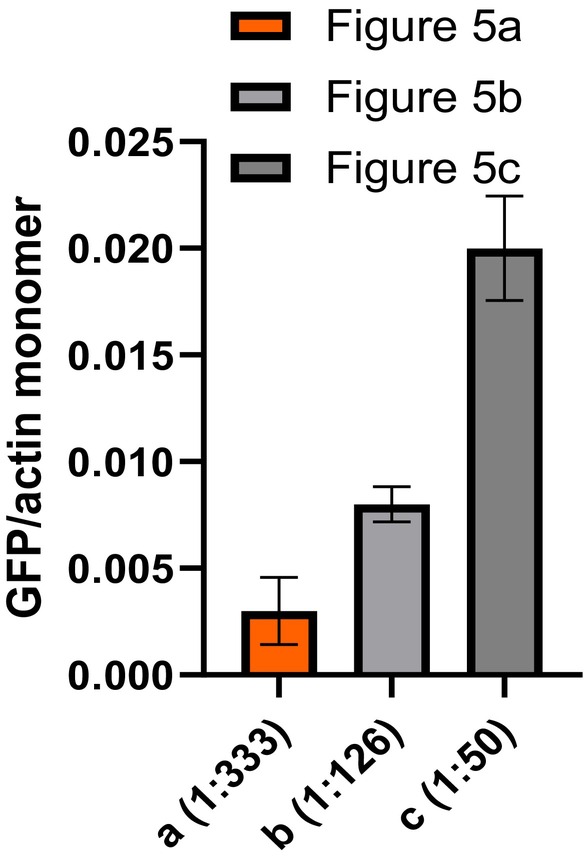
Quantification of myosin density on F‐actin based on panels (a–c) in Figure 5. Estimation shows almost one order of magnitude variability of the sub‐fragment 1 long (S1L) to actin monomer ratio (1:50–1:333) between the different experiments. This corresponds to average distances between subsequent cross‐linking sites of S1L to actin of 138–837 nm. Error bars represent standard error of mean for the mean ratio in each experiment based on two to three filaments analyzed. GFP, enhanced green fluorescent protein.

In view of the mentioned problems, we decided to work with higher density decoration of F‐actin as this yielded a more reliable and efficient cross‐linking of myosin motors with lower unspecific S1L surface adsorption, Figure 5. The cross‐linked S1L density (S1L to actin monomer ratio on filament) for each individual panel in Figure 5 is given in Figure 6, showing almost one order of magnitude difference in S1L density between experiments. The results also show that the cross‐linking density is relatively low on average with stoichiometric ratios between actin and S1L >50. As further discussed below, such a low density seems to be favorable for highest activity, similar to that of actin‐activated ATP turnover in solution (Huang et al., 1990).

### 3.4 Alexa 647—ATP time series and binding to cross‐linked S1L

**FIGURE 7:**
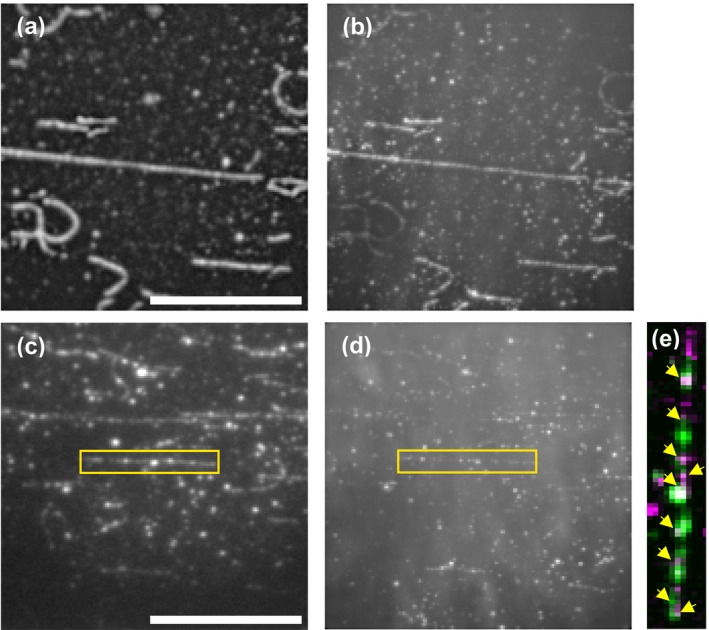
Visualization of sub‐fragment 1 long molecules cross‐linked to surface‐immobilized actin filaments. Visualization due to enhanced green fluorescent protein (eGFP) fluorescence and Alexa 647 ATP binding. (a) Visualization of eGFP bound to cross‐linked myosin in the absence of cold ATP. (b) Visualization of standard deviation time projection of Alexa 647 ATP in the absence of cold ATP for same region as in (a). (c–e). Visualization of eGFP (panel c) and corresponding standard deviation time projection of Alexa 647 ATP in the presence of 500 nM cold ATP (panel d). Yellow boxes highlight one F‐actin showing eGFP fluorescence and corresponding Alexa 647‐ATP fluorescence. Scale bars, 20 μm. (e) Merged image from yellow boxes after brightness adjustments and pseudo coloring of eGFP (green) and Alexa 647 ATP (magenta). Yellow arrows indicate co‐localization of eGFP and Alexa 647. The intensity of the merged pictures is normalized to further highlight co‐localized regions for visual clarification, procedure performed in image J. The Picture in E is rotated 90° clockwise from the yellow boxes in panels (c, d). See also Movies S5 and S6.

We obtained an overview of the spatial distribution of the Alexa 647 ATP interaction with cross‐linked S1L over 15‐min recordings using the ImageJ (Schneider et al., 2012) routine: *Image › Stacks › Z Projection › standard deviation*. This analysis, for example, in Figure 7, revealed hot‐spots for Alexa 647 ATP interactions with S1L along the actin filament. These hot‐spots, if co‐localizing with eGFP (see also Figure 7), were later in focus for our analysis of actin‐activated ATP turnover.

Initially the assay was performed in the absence of cold ATP (Figure 7a,b; Movie S5), but it was soon realized that this resulted in too frequent events that were not possible to distinguish as sm interactions with Alexa 647 ATP. This finding is consistent with the average distance between neighboring S1L cross‐linking sites on the actin filament in the range 137–837 nm (Figure 6), compared to the 3–3$\sqrt{2}$ pixels (.8–1.13 μm) width of the ROI. This corresponds to one to eight S1L molecules per ROI. Moreover, the inter‐S1L distances were smaller than the average value in several places along the filament due to non‐uniform decoration with cross‐linked S1L (Figure 5) giving even more S1L per ROI in these cases.

Because we were not able to decrease the S1L density along F‐actin (see above) we included different concentrations of non‐fluorescent, “cold” ATP in the assay solution to clearly distinguish individual Alexa 647 ATP–S1L interactions. As expected, including non‐fluorescent ATP resulted in appreciably fewer Alexa 647 ATP events occurring over time, enabling individual trace analysis. It was discovered that the addition of 500 nM of cold ATP, together with 5 nM Alexa 647‐ATP, most effectively allowed us to observe Alexa 647 ATP turnover by individual S1L molecules cross‐linked to actin (Figure 7c,d; Movie S6). Assays performed with Alexa 647‐ATP alone and in combination with cold ATP are compared in Figure 7.

### 3.5 Quantitative analysis of Alexa‐nucleotide on‐dwell times

**FIGURE 8:**
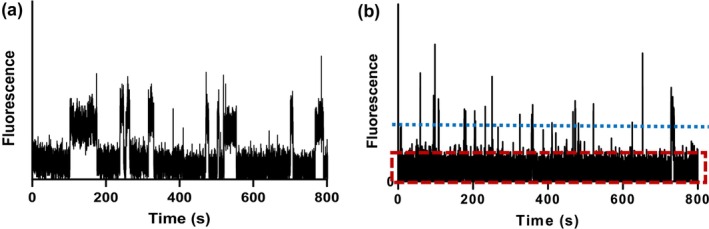
Measurements of basal myosin ATPase and actin‐activated ATPase. Representative time traces (15‐min recordings) from basal and actin‐activated ATPase achieved using sub‐fragment 1 long (S1L)‐enhanced green fluorescent protein (eGFP)‐Flag. (a) Representative time trace from expressed S1L‐eGFP‐Flag basal ATPase with the myosin motor domains immobilized via anti‐GFP antibodies to a nitrocellulose surface, time trace example from previous unpublished studies. (b) Representative time trace from cross‐linked actomyosin (this study). Red dashed lines enclose background noise, while dotted blue line shows the chosen threshold for data analysis of actin‐activated ATP turnover. Events of intensity below chosen threshold are not included in the analysis.

Time traces for events corresponding to either basal myosin Alexa 647 ATP turnover in the absence of actin or actin‐activated Alexa 647 ATP turnover are compared in Figure 8. The myosin motor domains for studies of basal ATP turnover were surface immobilized via anti‐GFP antibodies whereas they were cross‐linked to actin as described above for actin‐activated ATP turnover. It is clear (Figure 8) that dwell on‐times are significantly longer on average for myosin alone. Quantification of the cross‐linked actomyosin Alexa 647 ATP turnover (using 5 nM Alexa 647 ATP) was obtained in the presence of cold ATP (500 nM). This was found necessary to distinguish between individual Alexa 647 ATP on‐dwell events as outlined above.

**FIGURE 9:**
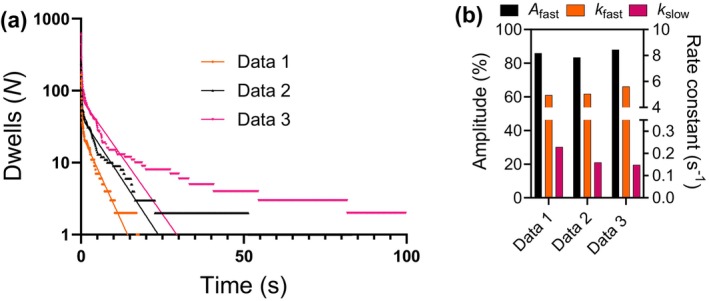
Cumulative frequency distributions of Alexa 647 ATP on‐dwell‐time events on sub‐fragment 1 long (S1L)‐enhanced green fluorescent protein (eGFP)‐Flag cross‐linked to F‐actin. (a) Frequency distributions with superimposed double‐exponential fits. (b) Rate constants (*k*_fast_ and *k*_slow_) and percentage amplitude (*A*_fast_) of fast exponential process, obtained from fittings of double‐exponential function to the cumulative frequency distribution in (a) (*R* ^2^ .96–.98, *N*_dwells_ are 166 (Data set 1), 236 (Data set 2) and 598 (Data set 3). Error bars refer to the 95% confidence interval in the regression analysis, the error bars are within the black drawn lines of the columns. The Data 1–3 are from independent experiments from three separate protein purifications on different days. For each protein purification, the experiment was performed once, and the data were extracted from one flow chamber. The experiments were performed within 24 h after the protein purification. Temperature 23 °C.

We analyzed time traces like those in Figure 8 for three different myosin preparations. It should be noted that the events in Figure 8b, observed in one given ROI, most likely represent intermittent binding of Alexa 647 nucleotide to several different (one to eight) S1 molecules (see above, Figure 6). Plotting of cumulative frequency distributions (Amrute‐Nayak et al., 2014; Usaj et al., 2021) of Alexa 647 ATP on‐dwell times from these analyses is illustrated in Figure 9 along with double‐exponential fits. Attempts to fit the data with three exponential processes were unsuccessful. We therefore used double‐exponential analysis only, which gave a fast rate constant with values in the range 4.95–5.60 s^−1^ and a slow rate constant in the range .15–.23 s^−1^. We attribute the fast process to actin‐activated ATPase and hypothesize that the slow process represents an unresolved mixture of unspecific Alexa 647 ATP binding outside the active site and basal ATPase activity.

The simulations below also suggest that the limited time resolution of our camera, by different mechanisms (Figure S3a,b), lead to a slightly underestimated rate constant of particularly the fastest process underlying the data. The relative amplitude of the slow and fast phase in the fits was in the range of 83.5%–88.0% and 12.0%–16.5%, respectively. Based on the theoretical analysis below and in Figure S2, the amplitude of the faster phases overestimates the relative number of molecules that underly this phase. Correction of the amplitudes according to equation 1 (see below) gives ranges of 11.9%–25.4% and 74.6%–88.6% for the fast and slow amplitudes, respectively. Thus, after correction/normalization according to Equation 1, the situation is inverse, suggesting appreciably more molecules underlying the slow than the fast events. As pointed out above, however, this correction is likely to underestimate the fast while overestimating the slow amplitude. Therefore, on the assumption that independent molecules are responsible for the slow and fast phase, we conclude that 11.9%–88% of the molecules exhibit fast kinetics whereas 12%—88.6% exhibit slow kinetics. Most likely, the true amplitudes are somewhere near the middle of the estimated ranges, that is, close to 50% for both fast and slow processes.

**FIGURE 10:**
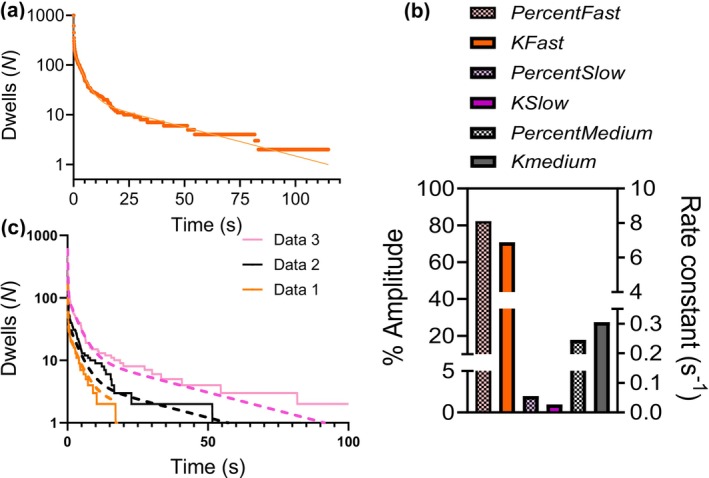
Cumulative frequency distribution of Alexa 647 ATP dwell‐time events on sub‐fragment 1 long cross‐linked to F‐actin. (a) Frequency distribution superimposed with fit of triple‐exponential function to the data. (b) Relative amplitudes (%) of the different phases (Checkerboard pattern; left vertical axis) and rate constants (right vertical axis) obtained from fitting to data in (a). Dwell‐on data from the three repetitions in Figure 9 were pooled (giving *N*_dwell_ = 1000). Error bars refer to 95% confidence interval obtained in the regression analysis. The error bars are hidden within the black drawn lines of the columns. Akaike's information criterion, 6862, *R* ^2^ (.987). (c) Reproduction of data from Figure 9, together with plots of triple‐exponential functions (without fitting) each using the parameter values from the fit to the pooled data in Figure 10a,b (after scaling to the total number of events, *N*_dwells_: 166–598). Note, faithful reproduction of the individual data sets in Figure 9 using one given set of parameter values obtained from fitting to the pooled data. Temperature 23 °C.

It is of interest to note that the rate constants and amplitudes from all three individual fits in Figure 9 are undistinguishable (Figure 9b) if one compares to the expected stochastic variability between random samples (Figure S4) for the number of dwell‐times studied. Thus, despite the apparent dissimilarity of the three different plots in Figure 9a (exaggerated by the plotting using a logarithmic vertical scale) we argue that each of these plots reflects identical underlying distributions. This accords with (i), the greater probability of capturing very rare slow events when a large total number of events are studied as for Data 3 (yet, only 7 out of 598 of these events in Figure 9a are >20 s) and (ii) the very large variability of the slow turnover rate constant between different Monte‐Carlo simulations with low *N* in Figure S4A. In view of these arguments, we pooled the experimental data from the three individual experiments in Figure 9 to increase the total sample size to *N* = 1000. Based on nonlinear comparison (using Graph pad Prism) between two phase‐ and three phase decay of the pooled data, we found that the pooled data were best fitted by a three‐phase exponential function suggested by a difference of 1800 in Akaike's information criterion (8662 vs. 6862) (see legend Figure 10). Such a fit gave 81.9%–82.6% fractional occurrence of a fast process characterized by a turnover rate of 6.76–7.00 s^−1^. This is higher than the rate constant of the fastest phase based on double‐exponential fits to individual experiments in Figure 9. It is comparable to the *k*_cat_ of 8.1 ± 1 s^−1^ (*n* = 3; mean ± SEM) measured from actin‐activated ATPase in solution in previous experiments under otherwise similar conditions, but using cold ATP rather than Alexa 647 ATP as substrate (Velayuthan et al., 2023). We also observed a slow phase with a rate constant of .03 s^−1^ and relative occurrence according to the fit of <2%. We attribute this phase to myosin heads exhibiting basal ATP turnover (Velayuthan et al., 2023). Such heads may be accidentally surface‐adsorbed outside the actin filaments or, for some reason, cross‐linked to actin in a configuration that is not accessible for productive cycling with actin binding. We now also observed a third phase characterized by rate constant of .29–.31 s^−1^ and a relative occurrence of 16%. We have previously (Usaj et al., 2021) attributed a similar phase in sm studies of skeletal muscle myosin to unspecific binding of ATP to the myosin motor domain outside the active site. If the latter interpretation is correct, it seems that cardiac myosin shows similar binding. Importantly, we could use the results from Figure 10a,b to further corroborate our assumption that all three data sets in Figure 9 represent identical underlying distributions. This view accords with the finding (Figure 10c) that *one* set of parameter values adequately reproduces all three individual data sets in Figure 9.

If we apply a correction procedure similar to that of Pilagov et al. (2023). Equation (1), which can be applied to more than two exponential processes (Pilagov et al., 2023), it suggests relative occurrences of the fast, intermediate and slow phases in our data of 9.3%, 43%, and 47%, respectively, quite different from the observed values. These corrected amplitudes, on the other hand, are, as shown above and in Figure S2, expected to underestimate the occurrence of the fast processes and overestimate the slow. Without going into any further quantitative detail, this suggests that the occurrence of the fast process is between 9.3% and 82.2% whereas the occurrence of the slow process is in the range of <2% to 47% and the intermediate process in the range of 16%–43%.

To summarize our experimental results, they show: (i) that a substantial fraction of the observed single myosin molecules turn over ATP at a rate similar to, or somewhat slower than actin‐activated ATPase in solution, but with a difference that can be accounted for by methodological issues (e.g., Figures S2 and S3), (ii) an intermediately fast process of Alexa‐nucleotide binding exists in cardiac myosin as previously found in skeletal muscle myosin and attributed to non‐specific binding to the motor domain outside the active site (Moretto et al., 2022; Usaj et al., 2021). In addition, our analysis of the experimental data taken together with Monte‐Carlo simulations below shows that it is essential to analyze many dwells of 1000 or more. This is important both to overcome stochastic variability in small samples (Figure S4) and for full and proper characterization of all exponential processes included in the data. Thus, all three exponential processes were revealed only after pooling data from the three individual experiments to give a total number of 1000 dwell times to analyze. This also shifted the fastest rate constant toward a higher value closer to the *k*_cat_‐value observed in actin‐activated ATPase in solution.

### 3.6 Monte‐Carlo simulations: Key factors in the analysis of single molecule time‐traces due to turnover of fluorescent ATP

**FIGURE 11:**
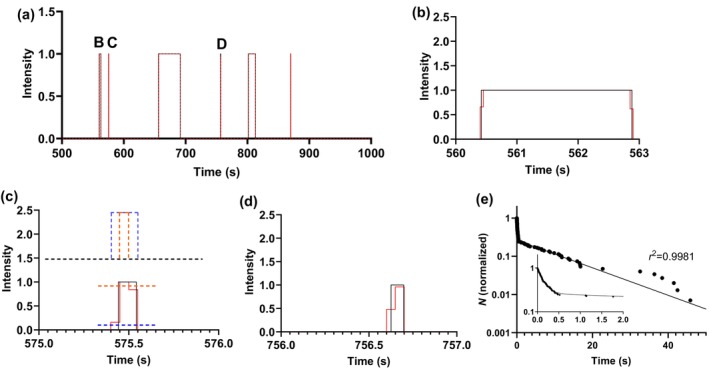
Monte‐Carlo simulation of Alexa 647 nucleotide on‐times and the effect of capturing the trace using 50 ms exposure times. (a) Time trace over 500 s with the simulated events in black and the simulated camera response in red. The letters B‐D refer to panels (b–d). (b) Expanded dwell‐on event B from panel (a). Same color coding as in (a). (c) Expanded dwell‐on event C from panel (a). Same color coding as in (a). Dashed lines, intensity threshold levels that may be used to select part of red trace for analysis. Inset (top), effect of different intensity threshold levels on digital trace used for input into frequency distribution. Blue: threshold .1 giving overestimation of event time. Orange: threshold .9 giving underestimation of event time. (d) Expanded dwell‐on event D from panel (a). Same color coding as in (a). (e) Cumulative frequency distribution obtained from simulation (*N* = 149 events; normalized) with similar underlying model as in panels (a–d). Line represents double‐exponential function fitted to the data by nonlinear regression (*r* ^2^ = .9981). Inset, data and fitted function at time ≤2 s. In the simulations, we assumed one fast myosin ATP turnover process with rate constant 8 s^−1^ and one slow process with rate constant .05 s^−1^, both processes attributed to one molecule each in the assumed region of interest studied. Alexa 647 ATP on‐rate constants .005 nM^−1^ s^−1^ .0025 nM^−1^ s^−1^ for molecule with fast and slow turnover respectively. (Alexa 647 ATP) set to 5 nM.

A Monte‐Carlo simulation time trace, corresponding to Alexa 647 ATP binding and turnover events is depicted in Figure 11. The data are simulated assuming that they arise from two different myosin molecules (details in the Supporting Information). In addition to illustrating the real events as predicted by our simulations we also illustrate the convolution of these events with the discrete sampling in 50 ms bins, due to the limited frame rate of our digital camera. To show the effect of the digitization more clearly, we expand the time scale for three events in Figure 11a given in Figure 11b–d. A cumulative distribution for a simulation with the same model parameter values as used in Figure 11a–d is given in Figure 11e, clearly revealing the two exponential processes corresponding to the different turnover rates of the two different molecules. This figure also illustrates the good fit of a double‐exponential function to the data. Please note, that these simulations do not cover all the complexities involved in analyzing real data. Noise from different sources, such as spatial variation in illumination and temporal variation in focus, is for instance not included.

Based on simulations like those in Figure 11 (see also the Supporting Information) we, however, elucidate several fundamental factors that affect the analysis of corresponding experiments (see above).

First, more fast than slow events are observed despite just one fast and just one slow molecule being assumed to be present in the simulations (Figure S2). This follows because the faster kinetics gives rise to more events per molecule and time during a given observation period simply due to more fast events fitting in during that period. This overestimation of the relative number of fast events has motivated correction of the observed amplitudes *A* ^obs,fast^ and *A* ^obs,slow^ obtained in fits to cumulative on‐time distributions as proposed by Pilagov et al. (2023):

(1) $$A^{\text{corr},\text{fast}}=A^{\mathrm{obs},\text{fast}}\;{k_{\mathrm{on}}}^{\text{slowl}}/{k_{\mathrm{on}}}^{\text{fast}},$$

where *A* ^corr,fast^ + *A* ^corr,slow^ = *A* ^obs,fast^ + *A* ^obs,slow^ = 1. Further, *k*_on_^slow^ = 1/*t*_on_^slow^ and *k*_on_^fast^ = 1/*t*_on_^fast^, where *t*_on_^slow^ and *t*_on_^fast^ are the average on‐dwell times for fluorescent ATP on myosin with slow and fast turnover, respectively.

We show in the supporting results (Figure S2), by a combination of analytical solutions and Monte‐Carlo simulations, that this correction is valid only under certain conditions. Under our present conditions, however, the correction of Pilagov et al. (2023) leads to an underestimation of the relative number of fast events. For that reason, we use the correction according to Equation 1 together with the observed amplitudes to define a lower and upper estimate, respectively of the fractional amplitude of the fast process. Naturally, with only two exponential processes assumed, this also defines upper and lower estimates of the relative amplitude of the slow process.

Another issue that is of interest to analyze is whether our frame rate with inter‐frame times of 50 ms is sufficient to faithfully quantify the fastest rate process in our records. Our Monte‐Carlo simulation‐based analysis shows that this is not necessarily the case (Figure S3A). Here, we use a threshold (see Figure 11C for definition) of 50% of the maximum intensity per event to allow us to attribute it to Alexa 647 ATP binding to myosin. Using this threshold value, it is shown in Figure S3A, that we would underestimate particularly the fast turnover rate constant by 25%, at a frame rate of 20 s^−1^ (as used in our experiments). This underestimation would be reduced by using a faster frame rate. Also, increasing the threshold level for a given frame rate would reduce the underestimation (Figure S3B). Conversely, the underestimation would be more severe if we select a lower threshold intensity value. The reason is that this will give an increase in average event duration (cf. Figure 11c) that is fractionally most important for short events. In view of the underlying reason for underestimated fast rates with low threshold levels, we also found that the underestimation is largely eliminated by subtracting half the frame time before applying nonlinear regression to the cumulative frequency distributions for threshold values below .5.

To summarize, the analysis in this paragraph suggests that (i) a frame rate of ≥20 s^−1^ should preferably be used to faithfully estimate the fastest rate constant associated with actin‐activated ATP turnover of cardiac β‐myosin under our experimental conditions and (ii) the threshold as defined above, should be higher than 50% of the average event intensity (as used here; Figure 8b).

Finally, our Monte‐Carlo simulation‐based analysis in Figure S4 elucidates the expected variability due to stochastic sampling that adds to uncertainties obtained in the regression analysis (which only constitutes a minor fraction of the entire error). The analysis in Figure S4 suggests that more than about 500 on‐dwell‐time events need to be analyzed to capture the true underlying parameter values with a reasonable probability. More exponential processes are likely to require even more events for reliable estimates of the underlying parameter values. This is partly illustrated by the experimental data below which suggest that more than 1000 events are needed in the case of three exponential processes.

## 4 DISCUSSION

### 4.1 Summary of the results and key implications

We have shown that human cardiac β‐myosin motor domain (S1L), cross‐linked to surface‐immobilized F‐actin, exhibits similar *k*_cat_ in our sm ATPase assay as estimated previously using an reduced nicotinamide adenine dinucleotide coupled bulk assay at the same temperature (Velayuthan et al., 2023). Whereas we use Alexa 647 ATP rather than ATP as substrate, we believe that the observed value of *k*_cat_ of 7 s^−1^ very well reflects the *k*_cat_ with ATP. First, a somewhat lower value observed in our sm data is expected from complexities due to limited camera frame rate (Figure S3). Second, the values of 7 and 8 s^−1^ are identical within the experimental uncertainty. Third convincing evidence has been presented that the kinetic properties are identical for ATP and Alexa 647 ATP when used as substrate for fast skeletal muscle myosin II both in the presence and absence of actin (Balaz et al., 2007).

The success of the new approach described here has numerous potential benefits. First, sm studies have the potential to convey information not readily obtained in ensemble studies, such as about heterogeneities between different molecules (e.g., with slow and fast ATP turnover) and the existence of certain non‐specific events (e.g., due ATP binding outside the active site). The issue with heterogeneities could not be strictly addressed here because the sm events that we studied in a given ROI (see above in connection with Figure 9b) reflect the pooled behavior of one to eight S1L molecules. However, the detection of non‐specific events is demonstrated by the three exponential processes found in Figure 10. The new actin‐linked assay for myosin can also be the starting point for a range of new interesting structural and functional studies as we consider further below.

However, the main reason that we developed the new sm method to obtain *k*_cat_ from actin‐cross‐linked myosin was to be able to perform kinetic actomyosin assays using minimal amounts of expressed S1L. Determination of *k*_cat_ of the actin‐activated ATPase in a bulk assay by fit to a hyperbolic equation of turnover rate versus actin concentration (>5 different), requires in total S1L purified from 10 to 20 plates of 60 mm diameter (Velayuthan et al., 2023) (for one replicate estimate). In these previous experiments, we used an S1L concentration of 50 nM (similar to that in Sommese et al., 2013) in a cuvette volume of 50–200 μL corresponding to >2.5 pmole or >.3 μg of S1L per actin concentration tested and in total ~2 μg of S1L. Expression and purification of such quantities is both rather expensive (requiring much transfection reagent) and labor intense, even when using the virus‐free method (Velayuthan et al., 2023). In contrast, estimation of *k*_cat_ using our sm method described here, only requires 5 μL out of a total elution volume of 200 μL purified from one single 60 mm plate for each replicate experiment. This corresponds to 1/80–1/40 of all S1L from one 60 mm plate of C2C12 cells, or 400–800 times less S1L than in the solution‐based assay requiring 2 μg of S1L. Whereas the amount of protein was too low to measure the concentration in the eluate from one 60 mm plate, the above calculations suggest that 5 μL elution buffer in our experiments contains about 2/(15 × 40) μg, that is, 3 ng or 24 × 10^−15^ mol S1L at a concentration of 5 nM.

The described sm method allows the determination of both *k*_cat_ and basal myosin ATP turnover with protein purified from one single 60 mm plate. In addition, considering that only a fraction of the S1L purified from this plate would be used, it will be possible to evaluate the effects of drugs using S1L from the same plate.

An interesting finding was the appearance of a third intermediate exponential process in the actin‐activated ATP turnover, seen when many events were studied. This process was complementing the processes corresponding to actin‐activated ATP turnover and basal ATP turnover. A similar intermediate process with a rate constant in the range .2–.5 s^−1^ has previously been found in sm studies of skeletal muscle myosin (Amrute‐Nayak et al., 2014; Usaj et al., 2021) as well as other myosins (Amrute‐Nayak et al., 2014). We recently (Moretto et al., 2022; Usaj et al., 2021) presented evidence that this process is attributed to non‐specific binding of ATP outside the active site, with the possible role to facilitate ATP binding (see also Zananiri et al., 2022) to the active site as well as contributing to multistep release of Pi.

### 4.2 Basis for assuming that cross‐linked myosin is representative for non‐cross‐linked myosin

Protease‐digestion, solution‐based ATP turnover assays, and structural studies (Duong & Reisler, 1989; Huang et al., 1990; Iwamoto et al., 2001; Mornet et al., 1981; Stein et al., 1985; Tawada & Kawai, 1990) suggest that the zero‐length EDC‐based cross‐linking of S1 to actin does not limit the myosin dynamics that is necessary to fulfill the actin‐activated ATP turnover cycle at normal rate. We have here verified this for the first time using a sm assay that also reveals other details of the process. We also report (to the best of our knowledge) the first study of human cardiac myosin in this regard. The previous studies discussed below were performed using skeletal muscle myosin. One previous study with cross‐linked myosin has been performed using preparations from the rat heart (ter Keurs et al., 2003).

It might seem surprising that a virtually unchanged rate of actin‐activated ATP turnover by myosin is possible after cross‐linking of myosin S1 to actin using a zero‐length cross‐linker. However, this can be understood from the idea that cross‐linking occurs via a flexible lysine rich loop in myosin (the loop 2 region between the 20 and 50 kD subdomains) (Yamamoto, 1990). In fast skeletal muscle myosin from the rabbit, the key sequence is ‐KKGGKKK (residues 637–643). Yamamoto (1990) reported that cross‐linking occurred mainly via the first and second lysine residues with a molar incubation ratio of actin to sub‐fragment 1 of 1:1. At higher molar ratio, giving lower density of myosin along actin, corresponding to conditions with higher average ATP turnover rate (seen at molar ratio of >2.7) (Huang et al., 1990), all five lysine residues were cross‐linked. The cross‐linking via the flexible loop 2 is one important basis for maintained myosin dynamics following cross‐linking. Considering the higher ATP turnover rate at low S1 density, it seems that the dynamics and/or reliable actin binding in a pre‐power‐stroke state during cycling also benefits from a high degree of cross‐linking via loop 2. In human cardiac β‐myosin, the homologous sequence is KGKGKAKK with as many lysines as in skeletal muscle myosin but spread out over nine instead of eight residues. To the best of our knowledge, the cross‐linking chemistry of cardiac myosin has not yet been studied.

There appear to be some differences and inconsistencies regarding turnover rates in previous actomyosin cross‐linking studies. This is partly attributed to the fact that S1 can be cross‐linked in different bound states based on the nucleotide in the active site (Arata, 1984). However, also myosin cross‐linked in rigor (with no ATP) appears to differ in properties between studies. As mentioned above, this may partly be attributed to variation in the molar ratio of myosin S1 on F‐actin during cross‐linking (Huang et al., 1990; Yamamoto, 1989, 1990) where the highest ATP turnover rate (similar to actin‐activated ATPase in solution) has been found at low density of myosin (as in our experiments; Figure 6). Yamamoto (1990) concluded that a cross‐linked single head myosin motor, interacts with F‐actin with considerable freedom of motion similar to the traditional S1‐actin complex in solution when the molar ratio of F‐actin to myosin S1 is ≥5. Huang et al. (1990) found qualitatively similar results but with optimal molar ratio of actin to myosin S1 of ≥2.7 times. In our study, we estimate, based on eGFP fluorescence, the cross‐linked actin to myosin S1 (S1L) density to correspond to a ratio of 50:1–333:1 (actin monomer:S1L), within the range where fastest actin‐activated ATP turnover is expected.

In addition to the actin‐myosin cross‐linking, another issue that is of relevance to consider is if the EDC cross‐linking of F‐actin to nitrocellulose maintains the dynamic properties of the F‐actin that exists in muscle cells. This issue is important in view of previous evidence for allosteric changes along actin filaments in response to various constraints (Galkin et al., 2010, 2012). In relation to both issues raised in this paragraph, our own results, with similar *k*_cat_ values as in solution ATPase studies of human cardiac myosin S1 further support the idea that neither the surface cross‐linking of F‐actin nor the cross‐linking of S1L to actin compromises the actin‐activated ATP turnover by myosin. Also, our findings of different affinities of HMM and S1 toward F‐actin (Greene, 1981), and the apparently cooperative binding of S1 to actin (consistent with previous findings) (Orlova & Egelman, 1997; Walker et al., 1994) point to a normally behaving actin filament, despite EDC cross‐linking to nitrocellulose.

### 4.3 Other methodological issues

In developing the present method, we initially attempted to achieve very low S1L density along F‐actin. However, we found that incubation with low S1L concentrations was associated with problems of S1L detachment from actin that we had not experienced in initial attempts using skeletal muscle HMM (data not shown). It is generally accepted that myosin sub‐fragment 1 has a lower affinity toward F‐actin than double‐headed HMM, in fact up to 1000 times lower (somewhat depending on the experimental conditions) (Greene, 1981). This lower affinity of sub‐fragment 1 may contribute to the problems of attaching just single‐headed molecules. We found that S1L detachment from actin occurred primarily at low S1L concentrations (where single fluorescent myosin molecules can be distinguished on F‐actin). This detachment also led to non‐specific surface adsorption of the detached molecules giving increased background fluorescence. The tendency for detachment as described, particularly at low S1L concentration, could be related to cooperative phenomena. S1 detachment has previously been observed in Cryo‐EM studies (Orlova & Egelman, 1997; Walker et al., 1994). The authors speculated that detachment of S1 from F‐actin in those cases (under conditions where the F‐actin is in excess over S1) was due to the cooperative removal of S1 by fluid forces. Another simple explanation is that ATP contaminants or other unwanted dissociation agents are present in the solution, which are enough to dissociate the actomyosin complex fully or partly, particularly at very low myosin concentrations. Independent of the cause, the issue as to why effective actin‐S1L cross‐linking at low [S1L] does not seem possible must be resolved. To overcome this issue, it is important to enable studies of true sm ATPase without overlapping cross‐linked S1L molecules in each studied ROI and without the need to use cold ATP to reduce the density of binding events for fluorescent ATP. This is not essential for obtaining *k*_cat_. However, it is required, for example, if there is a desire to evaluate individual, specific molecules either regarding heterogeneities or coupling of their product release to other molecular processes.

One further methodological issue to consider, is the possibility of cross‐linking between neighboring S1L molecules on actin that would lead to disturbed function. However, such events seem unlikely. First, the average calculated distance between neighboring S1L molecules on the actin filament was between 138 and 837 nm (Figure 6), far outside the reach of a zero‐length cross‐linker like EDC. Second, we would expect inter‐S1L cross‐linked complexes to produce unique traces consisting of mainly long dwell times representing slower/hindered myosin ATP turnover. Such traces were not detected arguing against this possibility.

Finally, methodological issues of importance are raised by our Monte‐Carlo simulations. It is shown in Figure S3, that it is important to select the threshold in the data analysis as close to the average intensity of longer events as possible, if the aim is to capture the true actin‐activated ATP turnover rate without risk of underestimation. Particularly, when comparing different conditions, for example, different drugs, drug versus control or different mutations it will be critical to select both the same threshold level and the same frame rate (preferably as high as possible) for each condition. If this is fulfilled, the Monte‐Carlo simulations including different number of events suggest that as many events (*N*) as possible should be included for each condition. If *N* > 500, it seems likely that changes of 25% and more due to effects of drugs, mutations, or other interventions would be detectable.

### 4.4 Limitations

As mentioned in the previous section, the data collected is not guaranteed to represent events from just one sm, but rather multiple myosin motors close to each other. We also mentioned that this limitation is of interest to amend for certain purposes.

With our setup we are limited by noise, for example, due to lack of auto‐focusing and other reasons for variation in light intensity with time (laser stability, instability of mechanical components, etc.). If these events can be overcome as seems likely with commercial systems, it should be straightforward to reduce the time between frames down to 10 ms. We found that this was possible also with our custom‐built system but not for routine use. One reason to increase time resolution is the underestimation of rate constants as fast as the cardiac actin‐activated ATPase (Figure S3) using the current time between frames of 50 ms.

One limitation in the present study is that the data analysis is done manually. This is inconvenient for analyzing larger quantities of data necessary for three‐phase exponential fitting and to minimize the effects of random variability (Figure S4). Manual data analysis could also contribute to human bias. To avoid such effects, we have imposed stringent criteria for including events in our analysis. Nevertheless, it would be of great value to introduce automatic analysis algorithms, for example, including machine learning or Bayesian inference to select appropriate events (Kinz‐Thompson et al., 2021; Smith et al., 2019; Tibbs et al., 2021; Wanninger et al., 2023).

### 4.5 Perspectives

We expect that the combination of virus‐free myosin expression, sm analysis using fluorescent ATP and the actin‐myosin cross‐linking approach will be useful for several purposes. One is drug screening where the methodology allows effects of drug candidates on the actin‐activated ATP turnover be tested on a wide range of mutations at relatively low cost. Another is basic mechanistic studies in which studies of many different mutations on actin‐activated ATP turnover would also be beneficial.

In the longer term, we expect to expand the utility of sm fluorescence in combination with virus‐free myosin II expression and the use of myosin cross‐linked to actin. Applications to structural studies, for example, to detect power‐stroke and other structural changes using sm‐FRET should be straightforward both because closely related ensemble studies have been reported (Muretta et al., 2015; Rohde et al., 2017) and because sm‐FRET has reached high level of maturity with studies in a range of proteins (Lerner et al., 2021). If the power‐stroke is detected at the same time as ADP‐release, using Alexa 647 nucleotide, this would be a first step toward sm transient kinetics characterization of the actomyosin ATP turnover and force generation. Here, one might also think of the possibility to detect release of Pi using phosphate‐binding proteins whose fluorescence increase upon Pi‐binding (Brune et al., 1994; Okoh et al., 2006; White et al., 1997). Whereas this is not straightforward one can think of developments that could make it feasible. If these developments will be fulfilled, the exciting possibility emerges to substitute stopped‐flow transient kinetics analysis with sm analyses requiring nanogram instead of microgram to milligram of proteins (Walklate et al., 2019). However, for this to be possible it will be important both to ensure that individual sm are faithfully probed, and that time resolution is improved as discussed above.

## 5 CONCLUSIONS

We have developed a new strongly miniaturized assay for studies of actin‐activated myosin ATP turnover, using EDC to cross‐link myosin to actin in functional form. Our results, based on binding events of single Alexa 647 nucleotide, show typical rates for both basal myosin II ATP turnover and activated‐ATP turnover by human cardiac β‐myosin but also a third exponential process that we, in previous studies of skeletal muscle myosin (Usaj et al., 2021), have associated with non‐specific binding of ATP outside the active site (Moretto et al., 2022; Usaj et al., 2021). We consider different aspects of the data analysis and methodology including its limitations, with suggestions for future improvements. Finally, we foresee that the methodology has the potential to be developed into a sm substitution for transient solution kinetics using stopped‐flow.

## AUTHOR CONTRIBUTIONS

Alf Månsson, Marko Ušaj, and Sven Tågerud designed research. Albin Berg, Marko Ušaj, and Lok Priya Velayuthan performed the experiments. Alf Månsson and Marko Ušaj coordinated the project and wrote the manuscript with contributions from the other authors. All authors reviewed the manuscript and approved the final version.

## CONFLICT OF INTEREST STATEMENT

The authors declare no conflicts of interest.

## Supporting information


**Data S1.** Supporting Information.

**Movie S1.** Alexa 647 phalloidin and biotin phalloidin labeled F‐actin attached to underlying nitrocellulose surface via streptavidin before addition of BSA. Note, rather firm attachment of F‐actin with slight movement. Size 122 × 122 μm^2^, 5 frames per second (shown at 10× this speed).

**Movie S2.** Alexa 647 phalloidin and biotin phalloidin labeled F‐actin attached to underlying nitrocellulose surface via streptavidin after addition of BSA (1 mg/mL). Note, increased detachment of filaments from the surface. Size 122 × 122 μm^2^, 5 frames per second (shown at 10× this speed).

**Movie S3.** After addition of BSA (1 mg/mL) to F‐actin (rhodamine‐phalloidin labeled) attached to underlying nitrocellulose surface via NEM‐HMM. Size 122 × 122 μm^2^, 5 frames per second (shown at 10× this speed).

**Movie S4.** After addition of BSA (1 mg/mL) to F‐actin (rhodamine‐phalloidin labeled) cross‐linked to underlying nitrocellulose surface. No detachment can be observed. Size 122 × 122 μm^2^, 5 frames per second (shown in real time).

**Movie S5.** S1L cross‐linked to F‐actin on surface but also S1L adsorbed directly on surface, in the presence of 5 nM Alexa 647‐ATP that intermittently binds to S1L. Recording for 1 minute corresponding to data in Figure 7b. Size 40 × 40 μm^2^, 19.3 frames per second (shown in real time).

**Movie S6.** Actomyosin ATPase. Time laps TIRF microscopy image sequence of Alexa 647‐ATP (5 nM, in grayscale pseudo coloring) in the presence of the cold ATP (500 nM) intermittently binding to S1L cross‐linked to F‐actin on the surface (F‐actin filament positions depicted with green dots). The ~5‐min video, underlying the data from Figure 7d, was accelerated ~5 times (from 19.3 fps to 100 fps) for better presentation. Note that Alexa 647‐ATP binds also to S1L adsorbed directly on the surface. Bar represents 5 μm.

## Data Availability

The data that support the findings of this study are available from the corresponding author upon reasonable request.

## Footnotes

1 FDA: U.S. Food and Drug Administration
