# Supplementary material for: Probing actin‐activated ATP turnover kinetics of human cardiac myosin II by single molecule fluorescence
Source: Cytoskeleton (Hoboken). 2024 Apr 16;81(12):883–901. doi: 10.1002/cm.21858 (PMC11615843; doi:10.1002/cm.21858)
Supplement: Supplementary file 1 — Data S1. Supporting Information. [file CM-81-883-s004.docx]

**Supporting Information**

**Probing actin-activated ATP turnover kinetics of human cardiac myosin II by single molecule fluorescence**

by

Albin Berg, Lok Priya Velayuthan, Sven Tågerud, Marko Ušaj* and Alf Månsson*

Department of Chemistry and Biomedical Sciences, Faculty of Health and Life Science, Linnaeus University, SE 391 82 Kalmar, Sweden

Correspondence to

Marko Usaj (marko.usaj@lnu.se)

Alf Månsson (alf.mansson@lnu.se)

**Supporting Methods**

Monte-Carlo simulations of single molecule events corresponding to one myosin molecule with fast and one with slow Alexa 647 ATP binding and turnover in a given region of interest. This corresponds to the following kinetic scheme.


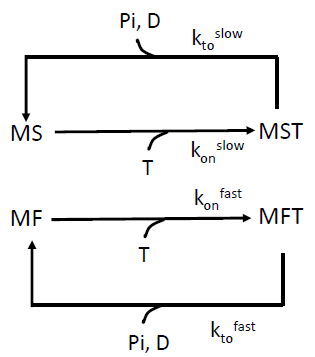


where MS and MF represent a myosin motor without bound nucleotide with slow and fast ATP (T) binding/turnover respectively. MST and MFT represent these myosin motors after binding of ATP. The rate constants of ATP binding are given by k_on_^slow^ and k_on_^fast^ whereas the rate constants of turnover, with release of ATP hydrolysis products ADP (D) and inorganic phosphate (Pi) are given by k_to_^slow^ and k_to_^fast^;

The simulations were performed according to the following protocol:

1. Initiation, setting the number of molecules in each of the states MS and MF equal to 1 but with no molecules in the states MST and MFT.

2. Calculation of the time delay for occurrence of the next transition according to the Gillespie algorithm^1^:

3. Selection of the actual event and updating the population of the different states according to the Gillespie algorithm:

4. Storage of all relevant time points in files for later analysis. Storage either of real times given by the simulations or digital times assuming a time frame of 50 ms. In the latter case an amplitude of 0.5 (an Alexa-647 ATP on event for more than 50 % of a 50 ms frame) was taken as a threshold for an event to be counted in a give frame.

5. Repetitions of the Gillespie steps according to 2 and 3 until pre-set simulation time period is reached.

6. Analysis of all simulated data as the analysis of experimental data.

**Supporting Results**

*NEM-HMM binding to Alexa 647 ATP*

Being encouraged by the NEM-HMM based attachment, we decided to proceed with single molecule actin-activated ATPase experiments. Actin filaments were attached to the surface via NEM-HMM while β− cardiac myosin sub fragment 1 were crosslinked to F-actin as described in the method section. Upon addition of assay solution containing 5 nM Alexa 647-ATP many spots were observed all over the chamber instead of co-localization with the actin filaments. Observed high background was attributed to binding of Alexa 647-ATP to NEM-HMM. This was confirmed in control experiments (**Fig. S1)** where, upon addition of Alexa 647-ATP (5 nM) to a chamber containing only NEM-HMM attached to nitrocellulose surface, trapping of labelled ATP was observed. The potential overlap and depletion of labelled ATP prevented detection of any actin activated ATPase activity.

**
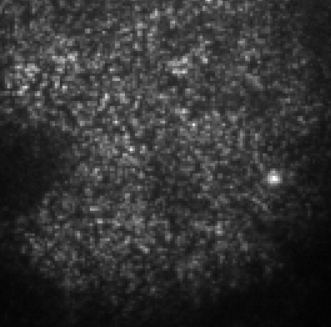
**

**
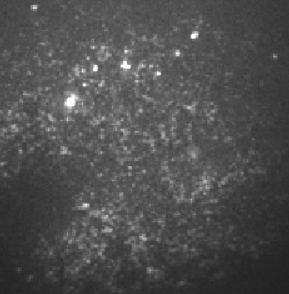
**


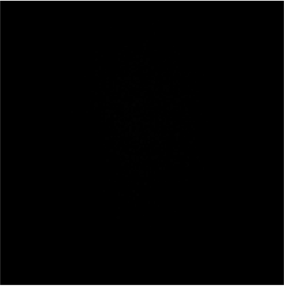


**Figure S1.** Unspecific binding of Alexa 647-ATP to NEM-HMM. **(A)** Time projection (10s) of Alexa 647-ATP fluorescence signal from a region where actin filaments were immobilized via NEM-HMM and subsequently crosslinked with S1L motors. **(B)** Time projection (10s) of Alexa 647-ATP from a region where only NEM-HMM was deposited **(C)** NEM-HMM region without addition of Alexa 647-ATP. Note no observed signal in **C** suggesting that the background in **A** and **B** was indeed the consequence of binding of Alexa 647-ATP to NEM-HMM, presumably to the active site.

*Correction of fast and slow amplitudes in double exponential distribution of Alexa-ATP on dwell times*

Myosin molecules with faster turnover give rise to more events per molecule and time during a given observation period simply due to more fast events fitting in during that period. Therefore, amplitudes obtained in fits to cumulative on-time distributions overestimate the number of molecules associated with fast events compared to those associated with slow events. In order to correct for that effect Pilagkov et al corrected the observed (A^obs^) relative amplitudes (fraction or percentage of all events). Here we follow the procedure of Pilagkov et al. by correcting the fast amplitude to A^obs,f^ using the observed on-times for the fast (rate constant k_on_^f^) and slow (rate constant k_on_^s^) processes as follows:

A^corr,f^=A^obs,f^ k_on_^s^/ k_on_^f^ (1)

Here, A^corr,f^ + A^corr,s^ = A^obs,f^ + A^obs,s^ = 1. Further, k_on_^s^ = 1/t_on_^s^ and k_on_^f^ = 1/t_on_^f^ where t_on_^s^ and t_on_^f^ are the average on dwell times for Alexa-647 ATP on myosin with slow and fast turnover, respectively. Using the amplitude correction in Eq. 1 the relative amplitude of the fast process become appreciably lower than the observed value. However, as demonstrated in Fig. S2, this now leads to an underestimation of the fast amplitude. The reason is that the correction is strictly valid only if t_on_^s^ + t_on_^f^ >> t_off_^s^ + t_off_^f^ where t_off_^s^ and t_off_^f^  is the average off dwell times when Alexa-647 ATP is not bound to myosin. However, this condition usually does not apply because t_off_^s^ + t_off_^f^ must be rather long (e.g. by keeping [Alexa-647 ATP] low) to allow observation of predominantly single Alexa-647 ATP binding events. Under such conditions, the appropriate amplitude correction would instead be obtained as follows:

A^corr,f^=A^obs,f^ (t_on_^f^  + t_off_^f^)/( t_on_^s^ + t_off_^s^). (2)

Using the correction according to equation 1 instead, would underestimate the fast process. However, in most real situations where both fast and slow processes exist simultaneously (in a given molecule or in a given observed ROI), t_off_^s^ and t_off_^f^ are not known exactly and cannot be readily estimated. Under these conditions, Equation 2 is not practically useful. Therefore, we instead use equation 1 to obtain a lower estimate of the fast amplitude from A^corr,f^ (and the associated highest estimate of the slow amplitude; 1- A^corr,f^) and take the value obtained in the fit, (A^obs,f^) as the upper estimate.

**Figure S2. Effects of the ratio between Alexa 647 ATP turnover rate constants and Alexa 647 ATP on rate constants for active site binding on relative amplitude of fast phase estimated from double exponential fits.** A. Average proportion of fast events calculated directly from model rate constants, for a range of combinations of values of k_on_^s^, k_on_^f,^ k_to_^s^ and k_to_^f^ and equal number of molecules with fast and slow kinetics. Proportions plotted against a ratio between the minnimum Alexa 647 ATP binding rate constant and sum of turnover rate constants. No Monte-Carlo simulations used for construction of this graph. Black symbols show fractional number of fast events associated with shorter time of each fast compared to slow event. Red symbols represent correction (normalization) as suggested by Pilagov et al. ^2^ (Eq. 1). B. Fractional fast amplitudes derived from Monte-Carlo simulations followed by double exponential fits to the data. Model parameter values with k_on_^s^ =0.0025 nM^-1^s^-1^, k_on_^f^ =0.005 nM^-1^s^-1^ for red and grey data set but k_on_^s^ =0.0010 nM^-1^s^-1^, k_on_^f^ =0.0020 nM^-1^s^-1^ for blue data set, with Alex 647 ATP taken as 5 nM. The values of the rate constants k_to_^s^ and k_to_^f^ indicated inside the figure. Each simulation assumes similar number of molecules with fast and slow kinetics and include 65 - 250 events. Three different data points for each rate constant combination (each color) result from three different simulation runs, each giving a given slightly different estimated set of parameter values due to random variability. Red, blue and grey circles represent data after normalization as suggested by Kad based on parameter values estimated from the simulated data followed non-linear regression analysis. Red, blue and grey crosses represent data after correction also taking into account estimated waiting times between on-dwells according to Eq. 2. Note, that the corrected fast amplitude using the latter correction is usually closer to the correct value (0.5) but with variability due to appreciable uncertainties in particularly the waiting times between events as estimated from the simulated data. Note, finally, the red data are for the same underlying model parameter values as the simulated time traces in Fig. 8 in the main paper. C. Correction without errors from fits and stochastic errors

**A**

**B**

**Figure S3. Effects of CCD camera time frame duration and threshold intensity level for selecting on-dwell-time data on parameter value estimation in double exponential fits.** A. Effects of time frame duration on estimated parameter values. The parameter values (fast and slow rate constants, relative fast amplitude) normalized to the values obtained in double-exponential fit to real data (without effects of discrete sampling). Threshold intensity amplitude for counting an event was set to 0.5 in these simulations. B. Effects of varied threshold intensity amplitude on the estimated parameter values normalized to parameters obtained in double-exponential fit to real data. All thresholds tested for a given simulated time trace. Underlying model parameter values in both A and B same as for simulations in Fig. 8 in the main paper.

**Figure S4. Variation due to stochastic effects in parameter values estimated after Monte-Carlo simulations and double exponential fits to samples of different sizes (7 different simulation runs per sample size).** Same underlying model parameter values as in Fig. 8 in the main paper. Interference by discrete sampling due to limited camera frame rate not included in these simulations.

**Supporting Movies (AVI format) Legends**

**Supporting movie 1.** Alexa-647 phalloidin and biotin phalloidin labeled F-actin attached to underlying nitrocellulose surface via streptavidin before addition of BSA. Note, rather firm attachment of F-actin with slight movement. Size 122x122µm^2^, 5 frames per second (shown at 10x this speed).

**Supporting movie 2.** Alexa-647 phalloidin and biotin phalloidin labeled F-actin attached to underlying nitrocellulose surface via streptavidin after addition of BSA (1mg/ml). Note, increased detachment of filaments from the surface. Size 122x122µm^2^, 5 frames per second (shown at 10x this speed).

**Supporting movie 3.** After addition of BSA (1mg/ml) to F-actin (Rhodamine-phalloidin labeled) attached to underlying nitrocellulose surface via NEM-HMM. Size 122x122µm^2^, 5 frames per second (shown at 10x this speed).

**Supporting movie 4.** After addition of BSA (1mg/ml) to F-actin (Rhodamine-phalloidin labeled) crosslinked to underlying nitrocellulose surface. No detachment can be observed. Size 122x122µ^2^, 5 frames per second (shown in real time).

**Supporting movie 5.** S1L cross-linked to F-actin on surface but also S1L adsorbed directly on surface, in the presence of 5nM Alexa 647-ATP that intermittently binds to S1L. Recording for 1 minute corresponding to data in Fig. 7B. Size 40x40 µm^2^, 19.3 frames per second (shown in real time).

**Supporting movie 6.** Actomyosin ATPase. Time laps TIRF microscopy image sequence of Alexa647-ATP (5 nM, in grayscale pseudo coloring) in the presence of the cold ATP (500 nM) intermittently binding to S1L cross-linked to F-actin on the surface (F-actin filament positions depicted with green dots). The ~5-minute video, underlying the data from Fig. 7D, was accelerated ~5 times (from 19.3 fps to 100 fps) for better presentation. Note that Alexa647-ATP binds also to S1L adsorbed directly on the surface. Bar represents 5 μm.

**Supporting References**

1. Gillespie, D.T. A general method for numerically simulating the stochastic time evolution of coupled chemical reactions. *Journal of computational physics* **22**, 403-434 (1976).

2. Pilagov, M., Heling, L., Walklate, J., Geeves, M.A. & Kad, N.M. Single-molecule imaging reveals how mavacamten and PKA modulate ATP turnover in skeletal muscle myofibrils. *J. Gen. Physiol.* **155** (2023).

3. Usaj, M., Moretto, L., Vemula, V., Salhotra, A. & Mansson, A. Single molecule turnover of fluorescent ATP by myosin and actomyosin unveil elusive enzymatic mechanisms. *Commun Biol* **4**, 64 (2021).
